# Bacterial and Fungal Adaptations in Cecum and Distal Colon of Piglets Fed With Dairy-Based Milk Formula in Comparison With Human Milk

**DOI:** 10.3389/fmicb.2022.801854

**Published:** 2022-03-23

**Authors:** Ahmed Elolimy, Fernanda Rosa, Patricia Tripp, Mohamed Zeineldin, Anne K. Bowlin, Christopher Randolph, Michael S. Robeson, Laxmi Yeruva

**Affiliations:** ^1^Arkansas Children’s Nutrition Center, Agricultural Research Service (ARS), United States Department of Agriculture (USDA), Little Rock, AR, United States; ^2^Department of Pediatrics, University of Arkansas for Medical Sciences, Little Rock, AR, United States; ^3^School of Veterinary Medicine, Texas Tech University, Amarillo, TX, United States; ^4^Department of Animal Medicine, College of Veterinary Medicine, Benha University, Banha, Egypt; ^5^Department of Microbiology and Immunology, University of Arkansas for Medical Sciences, Little Rock, AR, United States; ^6^Center for Translational Pediatric Research, Arkansas Children’s Research Institute, Little Rock, AR, United States; ^7^Department of Biomedical Informatics, University of Arkansas for Medical Sciences, Little Rock, AR, United States

**Keywords:** human milk, milk formula, distal gut, bacteria, fungi

## Abstract

Exclusive breastfeeding is recommended to newborns during the first 6 months of life, whereas dairy-based infant formula is an alternative nutrition source offered to infants. Several studies demonstrated that breastfed infants have a different gut bacterial composition relative to formula-fed infants. In addition, animal models have shown that human milk (HM)–fed piglets had a distinct intestinal bacterial composition compared with milk formula (MF)–fed piglets. However, the gut fungal composition and the interactions with the bacterial community in breastfed compared with formula-fed infants remain to be investigated. In an attempt to evaluate such differences, we used an animal model to perform a shotgun metagenomics analysis on the cecal and distal colon contents of neonatal piglets fed with pasteurized HM or a dairy-based infant formula (MF) during the first 21 days of life. At postnatal day 21 (PND 21), a subset of piglets from each diet group (*n* = 11 per group) was euthanized. The remaining piglets in each group were weaned to a solid diet and euthanized at PND 51 (*n* = 13 per group). Large intestine contents (i.e., cecum and distal colon) were subjected to shotgun metagenomics analysis. The differential taxonomic composition of bacteria and fungi and the predicted functional gene profiling were evaluated. *Bacteroidetes*, *Firmicutes*, *Proteobacteria*, and *Actinobacteria* are the most abundant bacterial phyla observed in piglets at PND 21 and PND 51. In the large intestine at PND 21 and PND 51, *Proteobacteria* phylum was significantly higher in MF-fed group, and species *Burkholderiales bacterium* of phyla was significantly higher in MF group relative to HM group. In addition, in HM group, several *Lactobacillus* spp. and *Bacteroides* spp. were higher relative to MF group in the large intestine at PND 21 and PND 51. Fungal genus *Aspergillus* was higher in MF, whereas *Malassezia* was lower relative to HM group. Persistent effects of the neonatal diets were observed at PND 51, where alpha- and beta-diversity differences were detected for bacterial and fungal species in the large intestine. Overall, our findings indicate that neonatal diet affects the large intestinal microbial community during the exclusive milk-feeding period, as well as after the introduction of the complementary food.

## Introduction

Humans contain trillions of microbial cells with different members such as bacteria and fungi (Sender et al., 2016). The gut microorganisms’ composition is likely impacted by several factors such as pH, oxygen levels, nutrients availability, birth mode, geographical location, and type of neonatal feeding [i.e., human milk (HM) vs. milk formula (MF)] (Hasan and Yang, 2019). Recently, The Environmental Determinants of Diabetes in the Young (TEDDY) study showed that, among all the factors, consumption of HM either exclusive or partial was the most significant factor in shaping the microbiota (bacteria) in infants (Stewart et al., 2018). Furthermore, several epidemiologic studies identified both acute (reduced necrotizing enterocolitis and bacterial sepsis) and long-term (reduced upper respiratory tract infection and ear infections) beneficial outcomes of HM compared with formula feeding during the infancy period (Hanson et al., 1985, 2003; Hahn-Zoric et al., 1990; Beaudry et al., 1995, 1996; Hanson and Korotkova, 2002). These results suggest that HM feeding results in positive health outcomes in infants.

Metabolic and immune health can be influenced by both diet and gut microbes. In fact, infants ≥ 4 weeks old fed with HM have higher abundance of fecal *Bifidobacteria* and *Bacteroides* than formula-fed infants (Balmer and Wharton, 1989; Mackie et al., 1999; Harmsen et al., 2000; Fanaro et al., 2003; Bezirtzoglou et al., 2011; Fallani et al., 2011). This is likely due to the bioactives such as HM oligosaccharides (HMOs), IgA, microRNAs, and milk microbiota, shaping early infant gut microbiota composition and function. Recently, it has been shown that lack of Bifidobacteria and particularly depletion of genes to utilize HMOs was associated with immune dysregulation. Interestingly, breastfed infants provided with *B. infantis* EVC001 strain showed decreased T helper 2 (Th2) and T helper 17 (Th17) response that is associated with inflammation and allergies (Henrick et al., 2021). Hence, the gut microbial population as an ecosystem likely plays a major role in infant early life immune function and likely impacts infection incidences and vaccine responses.

In addition to microbiota, most recent studies have suggested the role of fugal species in microbiota assembly and immune system development in the mouse model. Moreover, fungal dysbiosis was associated with atopy and asthma outcomes in children (Arrieta et al., 2015, 2018; Van Tilburg Bernardes et al., 2020a,b). Indeed, several studies have shown that dysbiotic microbiota (i.e., bacteria and fungi) is associated with immune system dysregulation with most common conditions such as colic, asthma, and allergies in children (Arrieta et al., 2015, 2018; Laforest-Lapointe and Arrieta, 2017; Rhoads et al., 2018; Van Tilburg Bernardes et al., 2020a,b; Amato et al., 2021). However, the majority of clinical trials have limitations associated with sample collection from different regions of the gastrointestinal tract in infants. Therefore, large animal models such as piglets have been used to study neonatal diet impact on gut, microbiota, and immune system (Jensen et al., 2001; Thymann et al., 2006; Comstock et al., 2014, 2017; Li et al., 2017; Rasmussen et al., 2017; Sun et al., 2019). Piglets also provide the opportunity to collect the samples from various regions of the gastrointestinal tract (Jensen et al., 2001; Thymann et al., 2006; Comstock et al., 2014, 2017; Li et al., 2017; Rasmussen et al., 2017; Sun et al., 2019). In addition, our published data from this animal model have shown an enhanced immune response by increasing the T-cell proliferation in the mesenteric lymph nodes of HM-fed versus MF-fed piglets (Miklavcic et al., 2018). Bioregional microbiota composition of large intestine showed an increased abundance of class *Bacteroidia* in HM-fed relative to MF-fed piglets at weaning and post weaning periods (Brink et al., 2019). However, the limitations with 16S rRNA gene analysis in the previous study did not identify the bacterial species.

We hypothesized that the distal gut microbial taxa and functional pathways exert communication between microbial taxa and the host, thus promoting metabolic and immune modulatory effects. To address this, large intestine luminal samples were subjected to shotgun metagenomics approach to determine the bacterial and fungal composition and predicted functional pathways at PND 21 and PND 51 of piglets fed with HM and MF diets.

## Materials and Methods

### Study Design and Sample Collection

The piglet study was conducted in accordance with the ethical guidelines for animal research approved by the Institutional Animal Care and Use Committee at the University of Arkansas for Medical Sciences (Protocol# 3727 and 3471). The experimental design and the detailed diet composition were reported previously (Miklavcic et al., 2018; Elolimy et al., 2020). Briefly, White Dutch Landrace Duroc male piglets at 2 days of age were randomly assigned into two groups (*n* = 24 per diet group) and fed with isocaloric diets: an HM group provided from Mothers’ Milk Bank of North Texas, TX, United States, or a cow’s milk-based formula group (MF; Similac Advance, Abbott Laboratories, Columbus, OH, United States). HM or MF diets were trained to drink from rubber nipples from day 2 of age until weaning at day 21 of age. The diets were formulated to meet National Research Council (NRC) guidelines for growing piglets (Yeruva et al., 2016). Solid starter pellets (Teklad 140608; Harlan Laboratories, Woodland, CA, United States) were introduced during the preweaning period from day 14 to day 21 of age. Piglets were fed with solid starter feed *ad libitum* without providing either HM or MF from day 21 to day 51 of age. At day 21 of age, 11 randomly selected piglets from each group were euthanized after anesthetization with a tight-fitting mask with a dose of 3–5% isoflurane and with oxygen between 0.8 to 1.5 L/min, followed by exsanguination, whereas the remaining piglets from each group (HM, *n* = 13; MF, *n* = 13) were euthanized at day 51 of age to collect cecum and distal colon contents. All samples were snap-frozen immediately in liquid nitrogen and stored at −80°C for subsequent metagenome analyses. This experimental design is illustrated in Supplementary Figure 1. Piglet growth data were published previously (Miklavcic et al., 2018); no growth differences were observed between HM diet– and MF diet–fed piglets.

### Cecum and Distal Colon Luminal Contents Metagenomics

DNA extraction and shotgun metagenomics sequencing: Cecum or distal colon contents (100 mg) were used for DNA isolation using the QIAamp Fast Stool Mini Kit Protocol (Qiagen, Germany) following the manufacturer’s standard instructions. Total DNA concentration and purity in each sample were measured using Nano Drop 2000c spectrophotometer (Thermo Fisher Scientific, United States). The extracted DNA was immediately stored at −80°C. DNA libraries were prepared using an Illumina Nextera XT kit (Illumina, Inc., San Diego, CA, United States) followed by shotgun sequencing performed on an Illumina NextSeq 500 platform with a 2 × 150–base pair, paired-end run. Raw sequence data files were demultiplexed and converted into FASTQ files using Casava v.1.8.2 (Illumina, Inc., San Diego, CA, United States).

Shotgun sequencing data processing and metabolic function profiling: The FASTQ sequence files were uploaded to the Metagenome Rapid Annotation Using Subsystems Technology (MG-RAST) version 4 webserver for taxonomic composition of bacteria and fungi to predict the functional gene profiles (Meyer et al., 2008, 2019). The RefSeq and SEED subsystem database tools implemented in MG-RAST were used to obtain the bacterial and fungal species and metabolic function profiles, respectively (Meyer et al., 2008, 2019). The sequences are publicly accessible at the MG-RAST webserver (https://www.mg-rast.org) under accession identification numbers mgm4916313.3 to mgm4916404.3. The web-based tool MicrobiomeAnalyst was used for microbiome statistical analyses and visual explorations as previously reported (Chong et al., 2020; Elolimy et al., 2020). The differences in taxonomic composition at the bacterial and fungal species and phylum levels between HM and MF samples were visualized using the default criteria for stacked bar/area plot option in MicrobiomeAnalyst (Chong et al., 2020). The differences in bacterial and fungal species and phylum relative abundances between the HM and MF groups were determined using the Mann–Whitney U group comparison test under the classical univariate analysis option in MicrobiomeAnalyst, where significance was determined at a *P*-value of ≤ 0.05 (Chong et al., 2020). Relative abundance of species at and above 0.1% was presented from distal colon lumen, and all the significant species were presented from cecal lumen. Data representing the β-diversity between the HM and MF groups were computed using principal coordinate analysis (PCoA) on the basis of non-phylogenetic Bray–Curtis distance metrics and the non-parametric multivariate analysis of variance test implemented in MicrobiomeAnalyst (Chong et al., 2020). The α-diversity was calculated in MicrobiomeAnalyst using observed species, Chao1, Shannon, and Simpson indices, where the Mann–Whitney pairwise comparison test was applied to detect significant differences between the two groups at a *P*-value of ≤ 0.05 (Chong et al., 2020). Statistical analysis of Metagenomic Profiles software (STAMP version 2.1.3) was used to assess and illustrate shifts in bacterial functions between HM and MF groups using the two-sided Welch’s *t*-test, where significance was determined at a *P*-value of ≤ 0.05 (Parks et al., 2014).

CAZymes analysis: The CAZymes (carbohydrate active enzymes) prediction was performed using dbCAN pipeline and classified on the basis of the CAZymes database. Briefly, preprocessed and quality-filtered sequences were *de novo*–assembled into contigs using SPAdes (Bankevich et al., 2012). The assembled SPAdes outputs were then annotated *via* Prokka (Seemann, 2014), followed by gene prediction using Prodigal (Hyatt et al., 2010). The CAZymes were annotated using the command line version of dbCAN server with e-value cutoff 1e-5 (Yin et al., 2012). The predicted CAZymes families [glycoside hydrolases (GHs), glycosyltransferases (GTs), polysaccharide lyases (PLs), carbohydrate esterases (CEs), and auxiliary activities (AAs)] were then assessed to evaluate the variety and abundance of the CAZymes families between HM- and MF-fed piglets. Statistical analyses were performed using JMP 13 software (SAS Institute, Inc., NC, United States). The least-squares mean of CAZymes families relative abundance between the HM and MF at different time points was compared using non-parametric Wilcoxon/Kruskal–Wallis Tests fitted in JMP 13 (SAS Institute, Inc.). *P*-values of < 0.05 were considered significant for all analyses.

## Results

### Formula Feeding Impacts Large Intestine Composition of Bacteria and Fungi at PND 21

α- and β-Diversity: The α-diversity measures for bacterial and fungal species showed no differences between the two groups (Supplementary Figures 2, 3) in the cecal and distal colon lumen. In addition, in cecal and distal colon lumen, no differences were detected in community structure as shown by β-diversity PCoA comparing MF-fed to HM-fed piglets (Supplementary Figures 2, 3).

**FIGURE 1:**
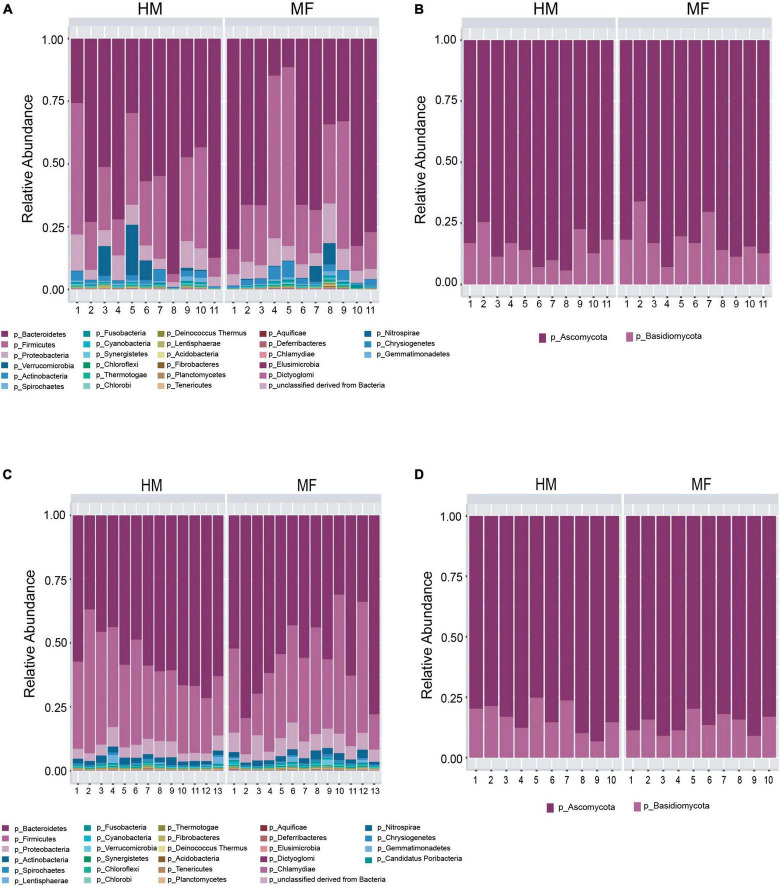
Taxonomic composition at phyla level at postnatal day 21 (PND 21) in male piglets fed with human milk (HM) or milk formula (MF) from day 2 until day 21 of age. **(A)** Cecal bacteria; **(B)** cecal fungi; **(C)** distal colon bacteria; **(D)** distal colon fungi.

Composition of microbial population in the cecum: In the cecal lumen, the top five bacterial phyla found at weaning were Bacteroidetes, Firmicutes, Proteobacteria, Verrucomicrobia, and Actinobacteria (Figure 1A and Supplementary Table 2). At the bacterial species taxonomic level, MF group had higher abundance of *Bacteroides thetaiotaomicron*, *Bacteroides uniformis*, *Bacteroides* sp. *D20*, *Bacteroides* sp. *1 1 14*, *Flavobacterium* sp., and *Streptomyces* sp. *e14* (Table 1 and Supplementary Data Sheet 1).

**TABLE 1:** Relative abundances of significant (*P* ≤ 0.05) cecum-associated bacterial and fungal species detected at weaning (i.e., day 21 of age) in male piglets fed with human milk (HM) or milk formula (MF) during the preweaning period from day 2 until day 21 of age.

Cecal luminal microbial species			
	Mean% abundance ± SEM		
Species	HM	MF	*P*-value
*Bacteroides thetaiotaomicron*	1.386 ± 0.125	2.539 ± 0.531	0.05
*Bacteroides uniformis*	0.389 ± 0.061	2.920 ± 0.810	0.01
*Bacteroides* sp. *D20*	0.356 ± 0.044	1.857 ± 0.483	0.02
*Bacteroides* sp. *1 1 14*	0.100 ± 0.012	0.248 ± 0.073	0.04
*Mycoplasma conjunctivae*	0.010 ± 0.003	0.004 ± 0.002	0.05
*Francisella philomiragia*	0.009 ± 0.001	0.007 ± 0.001	0.02
*Flavobacteriales bacterium ALC 1*	0.009 ± 0.002	0.005 ± 0.002	0.03
*Flavobacterium* sp.	0.007 ± 0.003	0.025 ± 0.006	0.01
*Lyngbya* sp. *PCC 8106*	0.005 ± 0.002	0.002 ± 0.000	0.05
*Nodularia spumigena*	0.005 ± 0.002	0.002 ± 0.001	0.02
*Borrelia bavariensis*	0.004 ± 0.002	0.002 ± 0.002	0.04
*Helicobacter mustelae*	0.002 ± 0.000	0.001 ± 0.000	0.03
*Bartonella tribocorum*	0.002 ± 0.000	0.001 ± 0.000	0.01
*Streptomyces* sp. *e14*	0.001 ± 0.000	0.002 ± 0.000	0.02
**Cecal luminal fungal species**			
*Yarrowia lipolytica*	4.609 ± 0.738	2.561 ± 0.564	0.05
*Laccaria bicolor*	2.305 ± 0.609	0.768 ± 0.348	0.04
*Ustilago maydis*	2.177 ± 0.549	4.481 ± 0.867	0.03
*Malassezia globosa*	1.793 ± 0.540	0.512 ± 0.214	0.05
*Pyrenophora tritici repentis*	1.152 ± 0.417	0.128 ± 0.128	0.05

*P-values were determined by Mann–Whitney test.*

The cecal fungal phyla found at weaning were Ascomycota and Basidiomycota (Figure 1B and Supplementary Table 2). At the fungal species level, the MF group had higher Ustilago maydis (Table 1 and Supplementary Data Sheet 2) relative to HM group. In contrast, MF-fed piglets had decreased abundance of *Yarrowia lipolytica*, *Laccaria bicolor*, *Malassezia globose*, and *Pyrenophora tritici repentis* in comparison with HM-fed piglets (Table 1 and Supplementary Data Sheet 2).

Composition of microbial population in the distal colon: In distal colon, the most abundant bacterial phyla found at weaning were Bacteroidetes, Firmicutes, Proteobacteria, Actinobacteria, and Lentisphaerae (Figure 1C and Supplementary Table 2). MF-fed piglets had higher Proteobacteria in distal colon relative to HM-fed piglets at weaning (Supplementary Table 3). At the bacterial species level, MF group had greater abundances for *Parabacteroides merdae*, *Burkholderiales bacterium 1 1 47* (Table 2 and Supplementary Data Sheet 3). Furthermore, MF-fed piglets had lower abundance of *Lactobacillus johnsonii*, *Bacteroides stercoris*, *Lactobacillus amylovorus*, *Lactobacillus reuteri*, and *Lactobacillus acidophilus* (Table 2 and Supplementary Data Sheet 3).

**TABLE 2:** Relative abundances of significant (*P* ≤ 0.05) distal colon-associated bacterial and fungal species detected at weaning (i.e., day 21 of age) in male piglets fed with human milk (HM) or milk formula (MF) during the preweaning period from day 2 until day 21 of age.

Distal colon luminal microbial species			
	Mean% abundance ± SEM		
Species	HM	MF	*P*-value
*Lactobacillus johnsonii*	2.277 ± 1.346	0.566 ± 0.210	0.03
*Bacteroides stercoris*	1.555 ± 0.398	0.508 ± 0.107	0.01
*Parabacteroides merdae*	0.336 ± 0.022	0.434 ± 0.040	0.05
*Lactobacillus amylovorus*	0.271 ± 0.093	0.022 ± 0.005	0.01
*Lactobacillus reuteri*	0.269 ± 0.049	0.116 ± 0.040	< 0.01
*Lactobacillus acidophilus*	0.241 ± 0.072	0.041 ± 0.009	0.01
*Bacteroides* sp. *1 1 14*	0.180 ± 0.070	0.067 ± 0.005	< 0.01
*Burkholderiales bacterium 1 1 47*	0.164 ± 0.050	0.425 ± 0.108	0.01
*Lactobacillus crispatus*	0.151 ± 0.045	0.034 ± 0.008	< 0.01
*Lactobacillus helveticus*	0.112 ± 0.032	0.020 ± 0.005	< 0.01
**Distal colon luminal fungal species**			
*Botryotinia fuckeliana*	3.596 ± 0.834	1.461 ± 0.412	0.03
*Ajellomyces capsulatus*	2.921 ± 1.233	0.562 ± 0.345	0.01
*Verticillium albo atrum*	0.337 ± 0.172	1.236 ± 0.353	0.05

*P-values were determined by Mann–Whitney test.*

The fungal phyla found in the distal colon at weaning were Ascomycota (83.48%) and Basidiomycota (Figure 1D and Supplementary Table 3). At the fungal species level, MF group had higher *Verticillium albo atrum* (*P* = 0.05) (Table 2 and Supplementary Data Sheet 5), whereas abundance of *Botryotinia fuckeliana* (*P* = 0.03) and *Ajellomyces capsulatus* (*P* = 0.01) was lower in MF group relative to HM group (Table 2 and Supplementary Data Sheet 4).

### Formula Diet Alters Microbial Function in the Large Intestine at PND 21

**FIGURE 2:**
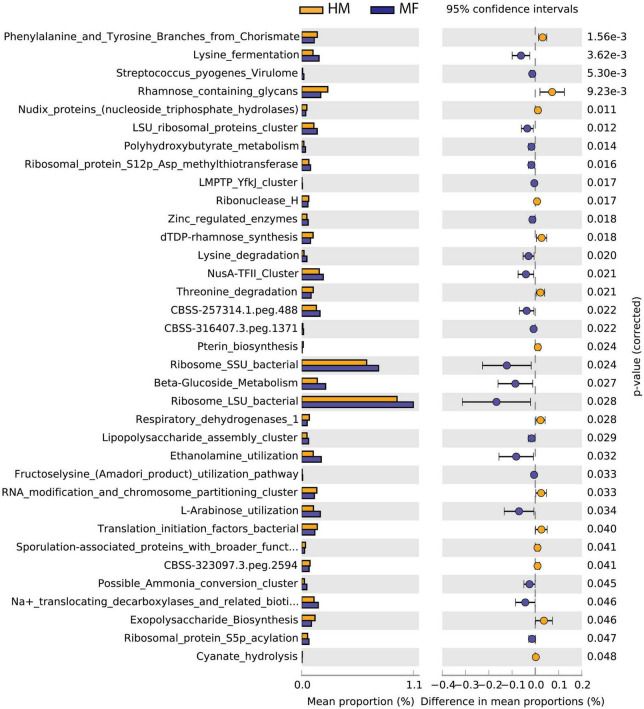
Significant differences in predicted metabolic functions (*P* < 0.05) for cecum bacterial gene profile detected at PND 21 in male piglets fed with HM or milk formula MF from day 2 until day 21 of age.

Cecal bacteria functional analysis (Figure 2) revealed that the cecum in MF group had a greater number of bacterial genes involved in lysine fermentation, Streptococcus pyogenes virulome, LSU ribosomal proteins cluster, polyhydroxybutyrate metabolism, and ribosomal protein S12p Asp methylthiotransferase (Figure 2 and Supplementary Data Sheet 5). On the other hand, the cecum in MF-fed piglets had a lower number of bacterial genes involved in phenylalanine and tyrosine branches from chorismate, rhamnose containing glycans, nudix proteins (nucleoside triphosphate hydrolases), ribonuclease H, and dTDP-rhamnose synthesis (Figure 2 and Supplementary Data Sheet 5).

**FIGURE 3:**
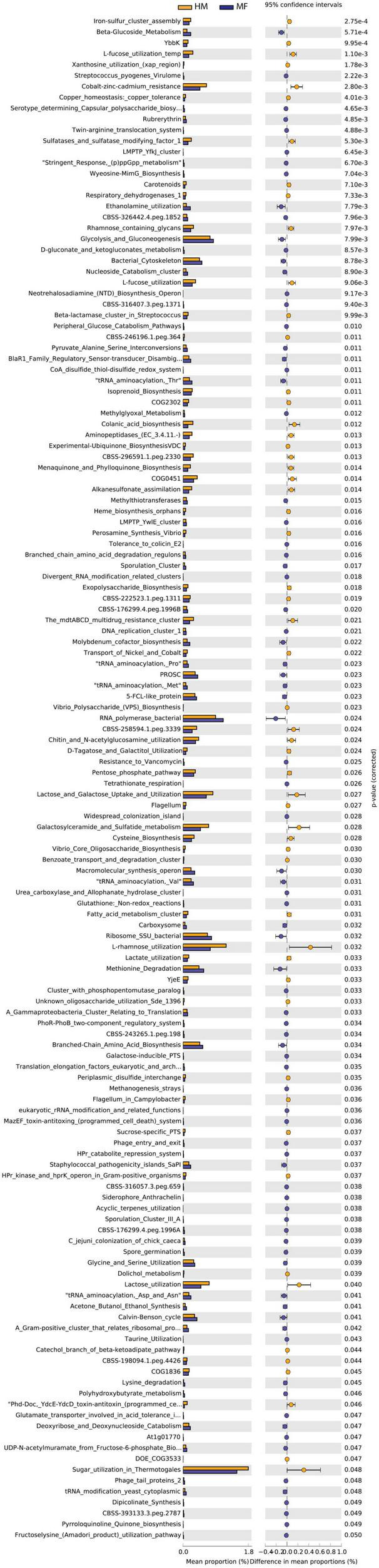
Significant differences in predicted metabolic functions (*P* < 0.05) for distal colon bacterial gene profile detected at PND 21 in male piglets fed with HM or MF from day 2 until day 21 of age.

Distal colon bacteria functional analysis (Figure 3) revealed that the distal colon in MF group had a greater number of bacterial genes involved in beta-glucoside metabolism, Streptococcus pyogenes virulome, serotype determining capsular polysaccharide biosynthesis in Staphylococcus, rubrerythrin, and twin-arginine translocation system (Figure 3 and Supplementary Data Sheet 6). On the other hand, the distal colon in MF-fed piglets had a lower number of bacterial genes involved in iron-sulfur cluster assembly, YbbK, L-fucose utilization temp, xanthosine utilization (xap region), and cobalt-zinc-cadmium resistance (Figure 3 and Supplementary Data Sheet 6).

### Formula Diet Impacts Diversity and Composition of Bacteria and Fungi in the Large Intestine at PND 51

α- and β-Diversity: Distal colon α-diversity analyses for bacterial species showed a higher Observed index in MF-fed relative to HM-fed piglets (Supplementary Figure 4). In the cecum, the α-diversity analyses for fungal species showed higher Shannon and Simpson indices for MF-fed piglets (Supplementary Figure 5). In the cecal lumen, MF feeding altered the community structure of fungal species as shown by β-diversity compared with HM-fed piglets (Supplementary Figure 5). In the distal colon, at the fungal species taxonomic level, MF-diet altered the fungal community structure as shown by β-diversity in comparison with HM-fed piglets (Supplementary Figure 5).

**FIGURE 4:**
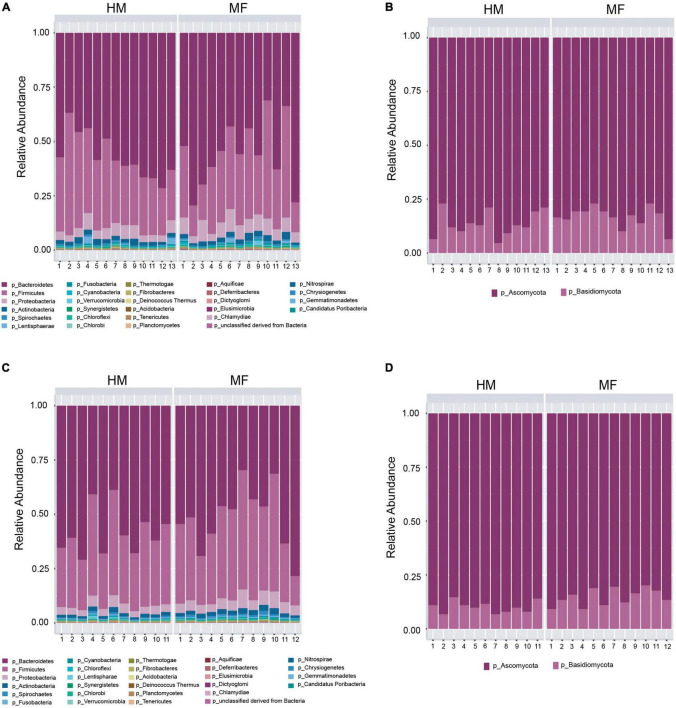
Taxonomic composition at phyla level at postnatal day 51 (PND 51) in male piglets HM or MF from day 2 until day 21 of age. **(A)** Cecal bacteria; **(B)** cecal fungi; **(C)** distal colon bacteria; **(D)** distal colon fungi.

Composition of microbial population in the cecum: The taxonomic composition of cecal lumen showed Bacteroidetes, Firmicutes, Proteobacteria, Actinobacteria, and Lentisphaerae (Figure 4A and Supplementary Table 4). MF group had higher Proteobacteria relative to HM group (Supplementary Table 4). At the bacterial species level, MF group had higher *Burkholderiales bacterium 1 1 47*, *Desulfovibrio vulgaris*, *Oribacterium* sp. *oral taxon 078*, *Syntrophomonas wolfei*, and *Geobacillus kaustophilus* (Table 3 and Supplementary Data Sheet 7). Interestingly, MF-fed piglets had decreased colonization of *Lactobacillus johnsonii*, *Bacteroides stercoris*, *Lactobacillus amylovorus*, *Lactobacillus reuteri*, and *Lactobacillus acidophilus* (Table 3 and Supplementary Data Sheet 7) relative to HM-fed piglets.

**TABLE 3:** Relative abundances of significant (*P* ≤ 0.05) cecum-associated bacterial and fungal species postweaning (i.e., day 51 of age) in male piglets fed with human milk (HM) or milk formula (MF) during the preweaning period from day 2 until day 21 of age.

Cecal luminal microbial species			
	Mean% abundance ± SEM		
Species	HM	MF	*P*-value
*Lactobacillus johnsonii*	2.270 ± 1.343	0.560 ± 0.211	0.03
*Bacteroides stercoris*	1.560 ± 0.399	0.513 ± 0.106	0.01
*Lactobacillus amylovorus*	0.272 ± 0.092	0.022 ± 0.005	0.01
*Lactobacillus reuteri*	0.270 ± 0.048	0.118 ± 0.038	0.01
*Lactobacillus acidophilus*	0.245 ± 0.074	0.037 ± 0.007	0.01
*Bacteroides* sp. *1 1 14*	0.177 ± 0.070	0.066 ± 0.005	0.00
*Burkholderiales bacterium 1 1 47*	0.164 ± 0.051	0.429 ± 0.108	0.01
*Lactobacillus crispatus*	0.149 ± 0.045	0.033 ± 0.008	0.01
*Desulfovibrio vulgaris*	0.146 ± 0.030	0.169 ± 0.017	0.05
*Lactobacillus helveticus*	0.114 ± 0.033	0.021 ± 0.005	0.00
**Cecal luminal fungal species**			
*Botryotinia fuckeliana*	10.049 ± 3.700	2.079 ± 0.773	0.01
*Schizosaccharomyces pombe*	3.119 ± 0.634	6.722 ± 0.833	0.01
*Nectria haematococca*	0.762 ± 0.286	2.010 ± 0.397	0.02
*Aspergillus niger*	0.624 ± 0.158	1.455 ± 0.298	0.03
*Malassezia globosa*	0.347 ± 0.192	2.148 ± 0.535	< 0.01

*P-values were determined by Mann–Whitney test.*

At PND 51 cecum fungal phyla found were Ascomycota and Basidiomycota (Figure 4B and Supplementary Table 4). At the fungal species level, MF group had higher levels of *Schizosaccharomyces pombe*, *Nectria haematococca*, *Aspergillus niger*, and *Malassezia globose* (Table 3 and Supplementary Data Sheet 10) but lower levels of *Botryotinia fuckeliana* relative to HM group (Table 3 and Supplementary Data Sheet 8).

Composition of microbial population in the distal colon: In the distal colon lumen, the bacterial taxonomic composition showed Bacteroidetes, Firmicutes, Proteobacteria, Actinobacteria, and Spirochetes (Figure 4C and Supplementary Table 5). MF-fed piglets showed higher abundance of Proteobacteria, Actinobacteria, Spirochetes, Cyanobacteria, Fibrobacteres, and Synergistetes (Figure 4C and Supplementary Table 5). At the bacterial species level, MF group had greater abundances for *Bacteroides capillosus*, *Faecalibacterium prausnitzii*, *Acidaminococcus fermentans*, *Slackia heliotrinireducens*, and *Fibrobacter succinogenes* (Table 4 and Supplementary Data Sheet 9). On the other hand, MF-fed piglets had decreased abundance of *Bacteroides stercoris*, *Bacteroides finegoldii*, *Pedobacter heparinus*, *Bacteroides* sp. *9 1 42FAA*, and *Capnocytophaga ochracea* (Table 4 and Supplementary Data Sheet 9).

**TABLE 4:** Relative abundances of significant (*P* ≤ 0.05) colon-associated bacterial and fungal species detected postweaning (i.e., day 51 of age) in male piglets fed with human milk (HM) or milk formula (MF) during the preweaning period from day 2 until day 21 of age.

Distal colon luminal microbial species			
	Mean% abundance ± SEM		
Species	HM	MF	*P*-value
*Bacteroides stercoris*	2.435 ± 0.581	0.448 ± 0.092	< 0.01
*Bacteroides capillosus*	1.159 ± 0.147	1.768 ± 0.197	0.04
*Faecalibacterium prausnitzii*	1.068 ± 0.127	2.015 ± 0.303	0.01
*Bacteroides finegoldii*	0.322 ± 0.028	0.231 ± 0.021	0.02
*Acidaminococcus fermentans*	0.212 ± 0.014	0.307 ± 0.041	0.03
*Pedobacter heparinus*	0.209 ± 0.019	0.145 ± 0.013	0.01
*Bacteroides* sp. *9 1 42FAA*	0.201 ± 0.013	0.147 ± 0.012	0.01
*Slackia heliotrinireducens*	0.188 ± 0.016	0.236 ± 0.016	0.05
*Capnocytophaga ochracea*	0.181 ± 0.025	0.110 ± 0.013	0.02
*Fibrobacter succinogenes*	0.160 ± 0.007	0.188 ± 0.010	0.04
*Bacteroides* sp. *1 1 14*	0.147 ± 0.049	0.061 ± 0.007	< 0.01
*Spirochaeta smaragdinae*	0.120 ± 0.027	0.211 ± 0.042	0.04
*Bifidobacterium longum*	0.113 ± 0.009	0.143 ± 0.009	0.03
**Distal colon luminal fungal species**			
*Yarrowia lipolytica*	7.920 ± 1.061	5.010 ± 0.564	0.04
*Saccharomyces cerevisiae*	2.844 ± 0.563	4.908 ± 0.623	0.04
*Ustilago maydis*	2.064 ± 0.354	3.732 ± 0.462	0.03
*Nectria haematococca*	1.004 ± 0.252	2.352 ± 0.328	0.01
*Aspergillus terreus*	0.725 ± 0.231	1.534 ± 0.277	0.04
*Laccaria bicolor*	0.502 ± 0.216	1.943 ± 0.464	0.03
*Malassezia globosa*	0.502 ± 0.200	1.176 ± 0.256	0.05

*P-values were determined by Mann–Whitney test.*

In distal colon, the taxonomic composition of fungi phyla showed Ascomycota and Basidiomycota (Figure 4D and Supplementary Table 4). MF group had greater Basidiomycota phyla and lower Ascomycota phyla in comparison with HM group. At the fungal species level, it is observed that MF group had higher abundance of *Saccharomyces cerevisiae*, *Ustilago maydis*, *Nectria haematococca*, *Aspergillus terreus*, *Laccaria bicolor*, and *Malassezia globose* (Table 4 and Supplementary Data Sheet 10) but lower abundance of *Yarrowia lipolytica* relative to HM group (Table 4 and Supplementary Data Sheet 10).

### Formula Diet Alters Microbial Function in the Large Intestine at PND 51

**FIGURE 5:**
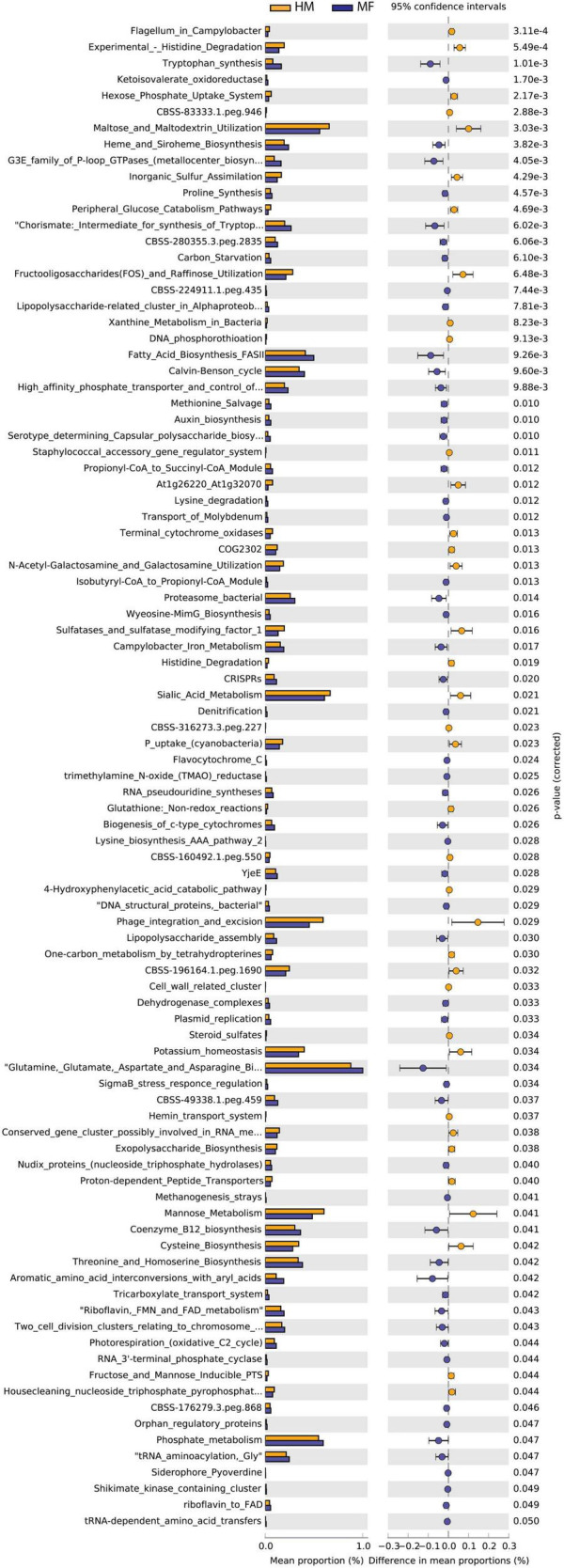
Significant differences in predicted metabolic functions for cecum bacterial gene profile detected PND 51 in male piglets fed with HM or MF from day 2 until day 21 of age.

Cecal bacteria functional analysis (Figure 5) revealed that the cecum in MF group had a greater number of bacterial genes involved in tryptophan synthesis, ketoisovalerate oxidoreductase, heme and siroheme biosynthesis, G3E family of P-loop GTPases (metallocenter biosynthesis), and proline synthesis (Figure 5 and Supplementary Data Sheet 11). On the other hand, the cecum in MF-fed piglets had a lower number of bacterial genes involved in histidine degradation, hexose phosphate uptake system, maltose and maltodextrin utilization, and inorganic sulfur assimilation (Figure 5 and Supplementary Data Sheet 11).

**FIGURE 6:**
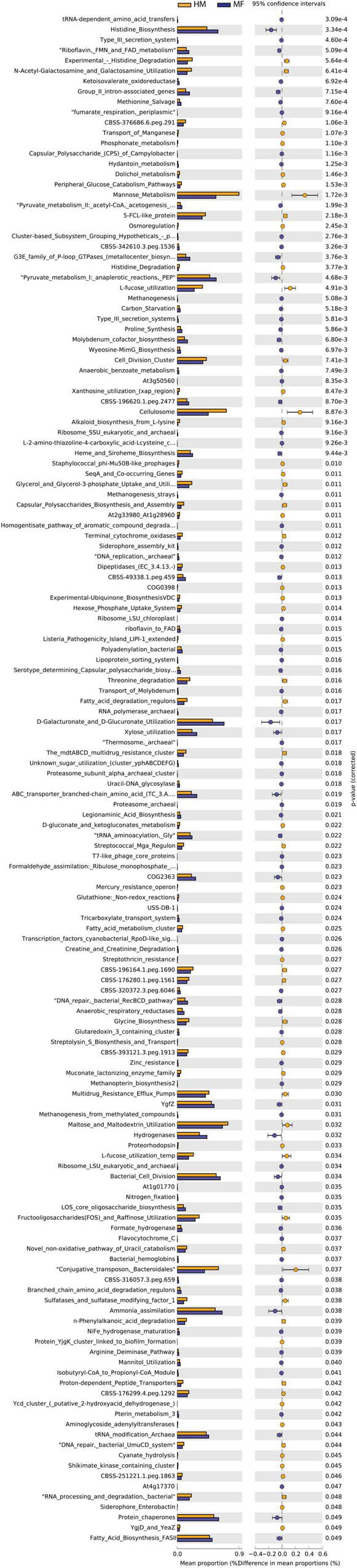
Significant differences in predicted metabolic functions (*P* < 0.05) for distal colon bacterial gene profile detected at PND 51 in male piglets fed with HM or MF from day 2 until day 21 of age.

Distal colon bacteria functional analysis (Figure 6) revealed that MF group had a greater number of bacterial genes involved in tRNA-dependent amino acid transfers, histidine biosynthesis, type III secretion system, riboflavin, FMN and FAD metabolism, and ketoisovalerate oxidoreductase (Figure 6 and Supplementary Data Sheet 12). On the other hand, the distal colon in MF-fed piglets had a lower number of bacterial genes involved in experimental-histidine degradation, N-acetyl-galactosamine and galactosamine utilization, transport of manganese, phosphonate metabolism, and dolichol metabolism (Figure 6 and Supplementary Data Sheet 12).

### Impact of Formula Diet on Expression of Carbohydrate-Active Enzymes

The relative abundance of the families of CAZymes was computed, and the resulting profiles are shown in Supplementary Figure 6 for the 46 most abundant families, and the enzymes were listed in Supplementary Data Sheet 13. The predominant CAZymes families across all samples were GT2 (9.4%), GT4 (8.04%) GH92 (4.413%), GH20 (4.413%), and GH29 (3.21%).

Composition of CAZymes families in the cecum: At day 21, the MF group had higher relative abundance of GH2 and GH32 relative to HM group. In contrast, MF-fed piglets had decreased abundance of GH25, GH95, GH13, GT51, GH57, and CE11 in comparison with HM-fed piglets (Supplementary Data Sheet 14). At day 51, the MF group had higher relative abundance of GT2_3, GT9, CE3, GH105, and GH106 relative to HM group. In contrast, MF-fed piglets had decreased abundance of GH20, CE1, GH2, GH33, CE4, GH13, GH16, and GH 109 in comparison with HM-fed piglets (Supplementary Data Sheet 14).

Composition of CAZymes families in the distal colon: At day 21, the MF group had higher relative abundance of CE1 and GT51 relative to HM group (Supplementary Data Sheet 14). In contrast, MF-fed piglets had decreased abundance of GH20, GH33, GH78, GH32, and GH106 in comparison with HM-fed piglets (Supplementary Data Sheet 14). At day 51, the MF group had decreased abundance of GH92, GH20, GH33, GH3, GH13, GH27 and GH 109 in comparison with HM-fed piglets (Supplementary Data Sheet 14).

## Discussion and Conclusion

Several prenatal (i.e., diet, environment, and obesity) and postnatal factors (i.e., delivery mode, and HM versus formula feeding) (Biasucci et al., 2010; Garcia-Mantrana and Collado, 2016; Stearns et al., 2017) impact infants gut ecology (i.e., bacteria and fungi). Of all the factors, HM has been shown to play a major role in gut microbiota composition and development (Stewart et al., 2018). However, majority of the studies assessed gut bacteria with limited data on fungal niche of HM-fed group. Moreover, several studies evaluated the HM fungal profile, but data on fungal species detected in intestinal tissues of animal models, and especially in infants during neonatal period are very limited. Our current study leveraged the samples from HM-fed piglet model and evaluated bacterial and fungal niche in the large intestine.

Data demonstrated that *Bacteroidetes*, *Firmicutes*, *Proteobacteria*, and *Actinobacteria* are the most abundant bacterial phyla observed in piglets at PND 21 and PND 51. In the large intestine at PND 21 and PND 51, *Proteobacteria* phylum was significantly higher in MF, confirming our previous 16S data (Brink et al., 2019). In addition, Lee et al. demonstrated higher amount of Proteobacteria in formula-fed infants in comparison with breastfed infants (Lee et al., 2015). To the best of our knowledge, this is the first report of the observation of higher abundance of Proteobacteria in formula-fed group relative to HM-fed group in a preclinical model. Moreover, this fits with previously published clinical study, thus offering an opportunity to study future mechanistic questions in a model where gut and several tissues can be collected in comparison with infant studies that are limited for various sample collection due to ethical limitations. Proteobacteria have been associated to inflammatory conditions and gut dysbiosis (Sim et al., 2010; Mukhopadhya et al., 2012). Specifically, *Burkholderiales bacterium* species of this phylum was significantly higher in the MF group relative to HM-fed piglets in our study. *Burkholderiales* are gram-negative bacteria and previously were shown to increase with consumption of western style diet for 12 weeks (Volynets et al., 2017). In addition, the same bacteria were higher in low-IgE asthma subjects relative to high-IgE asthma group (Wang et al., 2021). We speculate that the formula feeding–associated changes likely impact health outcomes, and this requires future translational and mechanistic studies to fully determine the impact of these taxonomic changes on the gut and immune system.

Novel finding in our model is that HM group had higher abundance of several *Lactobacillus* spp. and *Bacteroides* spp. relative to MF group in the large intestine at PND 21 and PND 51. Previously, it was demonstrated that HMOs are utilized by these species, suggesting the HM diet impact on these species colonization (Walsh et al., 2020). *Lactobacillus johnsonii* species has been shown to ameliorate intestinal and systemic inflammatory immune response in a *Campylobacter jejuni* infection model (Bereswill et al., 2017). *Lactobacillus* species have also been shown to produce indole derivatives that activate aryl-hydrocarbon receptor in CD4 + T cells and promote double positive intraepithelial lymphocytes (IELs) (Cervantes-Barragan et al., 2017). Interestingly, from the same piglet model, we have reported higher levels of indole derivatives in the large intestine of HM group relative to MF (Rosa et al., 2020), suggesting the likely role for this *Lactobacillus* spp. in maintaining higher levels of indoles in HM. Lactobacillus species have been suggested as probiotics for various positive health outcomes such as modulation of proinflammatory cytokines (Galdeano and Perdigon, 2004; Vinderola et al., 2005) increase IgA expression in the gut (Maldonado Galdeano et al., 2011), improve allergic inflammation (Velez et al., 2015), and likely function as adjuvant with cell wall components to induce stronger immune response (Lemme-Dumit et al., 2021). In fact, in this model, we have shown stronger immune response to vaccination (Miklavcic et al., 2018), further suggesting the beneficial effects of microbiota shaped by HM feeding. Moreover, previously, it was demonstrated that breastfed infants or infants fed with formula supplemented with HMOs (2′-FL) have shown lower inflammatory cytokine profile in comparison with formula-alone–fed infants (Goehring et al., 2016). In addition, data from monkey model demonstrated lower *Lactobacillus* genera in formula-fed group and higher inflammatory cytokine levels (O’sullivan et al., 2013). In addition, our data from the piglet model demonstrated that MF diet might impact intestinal inflammation and tight junctions and might suppress pathogen recognition (Elolimy et al., 2020). Together, high abundance of *Lactobacillus* spp. observed in HM-fed group might be protective for gut, augment immune response, and protect from infection during the infancy period. However, future mechanistic studies have to be carried out to directly link the microbiota changes to health outcomes.

Furthermore, the IELs play a role in protecting the intestinal tract from pathogens (Zhou et al., 2019). In addition, *Bacteroides* spp. have been shown to promote T-regulatory cell development (Round and Mazmanian, 2010; Round et al., 2011) and primary synthesizers of Vitamin K and help maintain intestinal homeostasis with short chain fatty acid production (Zafar and Saier, 2021). Likely, the HM bioactives play a major role in maintaining the gut bacterial colonization in HM group. Together, these data suggest that beneficial bacterial abundance is higher in HM-fed piglets, whereas gram-negative bacteria that were associated with inflammation and asthma outcomes were higher in MF-fed piglets. Our group previously reported a higher abundance of *Bacteroides* in the feces of HM-fed piglets relative to the formula-fed group (Miklavcic et al., 2018). In line with these observations, we detected greater abundance of GHs potentially expressed by *Bacteroides vulgatus* in the cecal contents of HM-fed piglets relative to MF-fed group at PND 21 (Rosa et al., 2021). Similarly, in the current study, the predicted metabolic function analysis for the bacterial profile showed an enhancement for the rhamnose-containing glycans pathway in the lumen of cecum of the piglets fed with HM relative to the MF-fed group at PND 21 (Figure 2). Peptides within this pathway are common enzymes involved in the metabolism of carbohydrates (Marcobal and Sonnenburg, 2012; Dewulf et al., 2013); it is plausible to speculate that milk glycan such as HMOs can promote the gut colonization of beneficial bacteria. Furthermore, from Cazy analyses, more number of CAZymes abundance was observed in HM-fed relative to formula-fed (Supplementary Figure 6). The relevance of these enzymes abundance in HM has yet to be determined. In addition, questions that remain unanswered including which components of bacteria or bacterial products could be promoting the health benefits in HM-fed versus formula-fed group are yet to be determined.

*Ascomycota* and *Basidiomycota* are the dominant fungal phyla observed at PND 21 and PND 51. These findings are in accordance with the CHILD cohort study, which reported *Basidiomycota* and *Ascomycota* as the dominant fungal phyla detected in the HM (Moossavi et al., 2020). *Aspergillus* spp. were higher in MF group relative to HM group at PND 51. Most-recently, *Aspergillus* was shown to positively associate with increased adiposity and weight gain (Mar Rodriguez et al., 2015). In addition, increased indoor *Aspergillus* was associated with increased incidence of rhinitis in later life (Behbod et al., 2015). In our study, the yeast *Malassezia* spp. were higher in HM-fed piglets at PND 21 but lower at PND 51 in comparison with MF-fed piglets. *Malassezia* spp. were observed in HM-fed piglets, possibly seeding the gut at PND 21 but not at PND 51 (Boix-Amoros et al., 2019; Heisel et al., 2019; Moossavi et al., 2020). A comparative approach evaluated the gut bacterial and fungal dysbiosis on the basis of fecal samples of 3-month-old infants from the CHILD and ECUAVIDA cohorts. This comparison showed an association between gut microbiome early life and the development of atopic wheezing in the childhood (Arrieta et al., 2018). An increased abundance of the yeast *P. kudriavzevii* in the feces was associated with the development of atopic wheeze at 5 years of age (Arrieta et al., 2018). Together, in the published studies and our findings, the fungal profile observed in the current study is derived from the HM feeding, and other yeasts might play a role in the infants’ susceptibility to diseases in early life. However, data are limited on fugal profile of HM-fed versus MF-fed; thus, future studies are needed to address this knowledge gap.

The piglet model used in this study has some limitations, which might have introduced some variation in the findings. The HM fed to the piglets was pasteurized pool from 2 to 12 months of lactation. In addition, piglets were enrolled in the trial at 2 days of age by which the colostrum quality and amount consumed are unknown. We acknowledge that such limitations might have played a role in the microbial profile of the distal gut at PND 21. However, the fact that animals from both diets were maintained in a controlled environment (housed at the vivarium), had similar growth, and both dietary groups consumed isocaloric diet composition prompted to further evaluate our results on the basis of the neonatal diet offered to the piglets.

Overall, gut fungal composition and function have been far less studied, especially, in early life. However, it is not surprising to think that interkingdom communication occurs between bacteria and fungi in the gastrointestinal tract. For example, syntrophism has been shown previously where methanogens consume H2, which helps *Ruminocococcus* spp. to produce more ATP from the same substrate (Stams and Plugge, 2009). Furthermore, less is known about co-existence of bacteria and fungi and association of core set of species from these taxa in health outcomes. Thus, future studies are needed to understand the bacterial and fungal composition and their association to health outcomes in infants.

## Data Availability Statement

The original contributions presented in the study are publicly available. The datasets can be found at Metagenome Rapid Annotation Using Subsystems Technology (MG-RAST) ver. 4 webserver under accession identification numbers mgm4916313.3 to mgm4916404.3.

## Ethics Statement

The animal study was reviewed and approved by University of Arkansas for Medical Sciences.

## Author Contributions

LY conceptualized the study. AE conducted data analyses and statistical analysis. PT contributed to data analyses. AB conducted the animal trial. CR performed samples sequencing. MR conducted data analyses. FR, AE, and LY interpreted the data and wrote the manuscript. AE and LY have the primary responsibility for the manuscript. All authors revised the manuscript draft and agreed to the published version.

## Conflict of Interest

The authors declare that the research was conducted in the absence of any commercial or financial relationships that could be construed as a potential conflict of interest.

## Publisher’s Note

All claims expressed in this article are solely those of the authors and do not necessarily represent those of their affiliated organizations, or those of the publisher, the editors and the reviewers. Any product that may be evaluated in this article, or claim that may be made by its manufacturer, is not guaranteed or endorsed by the publisher.

## Supplementary Material

Supplementary Figure 1 Flowchart of the experimental design used in this study. Piglets were randomly assigned to consume human milk or an isocaloric dairy milk–based formula, trained to drink from rubber nipples and were fed 1.047 MJ kg^–1^ d^–1^ of either human milk or milk formula. Piglets were fed every 2 h in the first week of the study, followed by every 4 h in the second week, and every 6 h in the third week through day 21. Solid “starter pig food” was slowly introduced at day 14 until day 21 and all piglets had transitioned completely to an *ad libitum* solid diet (Teklad diet, TD 140608; Harlan) at day 21. Piglet weights and diet consumption were recorded daily.

Supplementary Figure 2 Bacterial species at PND 21 in piglets fed with human milk (HM) or milk formula (MF). **(A)** Cecal alpha diversity represented by Observed, Chao1, Shannon, and Simpson indices. **(B)** Distal colon alpha diversity represented by Observed, Chao1, Shannon, and Simpson indices. **(C)** Cecal beta diversity determined by principal coordinate analysis (PCoA). **(D)** Distal colon beta diversity determined by PCoA.

Supplementary Figure 3 Fungal species at PND 21 in piglets fed with human milk (HM) or milk formula (MF). **(A)** Cecal alpha diversity represented by Observed, Chao1, Shannon, and Simpson indices. **(B)** Distal colon alpha diversity represented by Observed, Chao1, Shannon, and Simpson indices. **(C)** Cecal beta diversity determined by principal coordinate analysis (PCoA). **(D)** Distal colon beta diversity determined by PCoA.

Supplementary Figure 4 Bacterial species at PND 51 in piglets fed with human milk (HM) or milk formula (MF). **(A)** Cecal alpha diversity represented by Observed, Chao1, Shannon, and Simpson indices. **(B)** Distal colon alpha diversity represented by Observed, Chao1, Shannon, and Simpson indices. **(C)** Cecal beta diversity determined by principal coordinate analysis (PCoA). **(D)** Distal colon beta diversity determined by PCoA.

Supplementary Figure 5 Fungal species at PND 51 in piglets fed with human milk (HM) or milk formula (MF). **(A)** Cecal alpha diversity represented by Observed, Chao1, Shannon, and Simpson indices. **(B)** Distal colon alpha diversity represented by Observed, Chao1, Shannon, and Simpson indices. **(C)** Cecal beta diversity determined by principal coordinate analysis (PCoA). **(D)** Distal colon beta diversity determined by PCoA.

Supplementary Figure 6 CAZymes percent abundance in piglets fed with human milk (HM) or milk formula (MF). **(A)** Cecal abundance at PND 21. **(B)** Cecal abundance at PND 51. **(C)** Colon abundance at PND 21. **(D)** Colon abundance at PND 51.
