# Supplementary figures and images for: Bacterial and Fungal Adaptations in Cecum and Distal Colon of Piglets Fed With Dairy-Based Milk Formula in Comparison With Human Milk
Source: Front Microbiol. 2022 Mar 23;13:801854. doi: 10.3389/fmicb.2022.801854 (PMC8989072; doi:10.3389/fmicb.2022.801854)

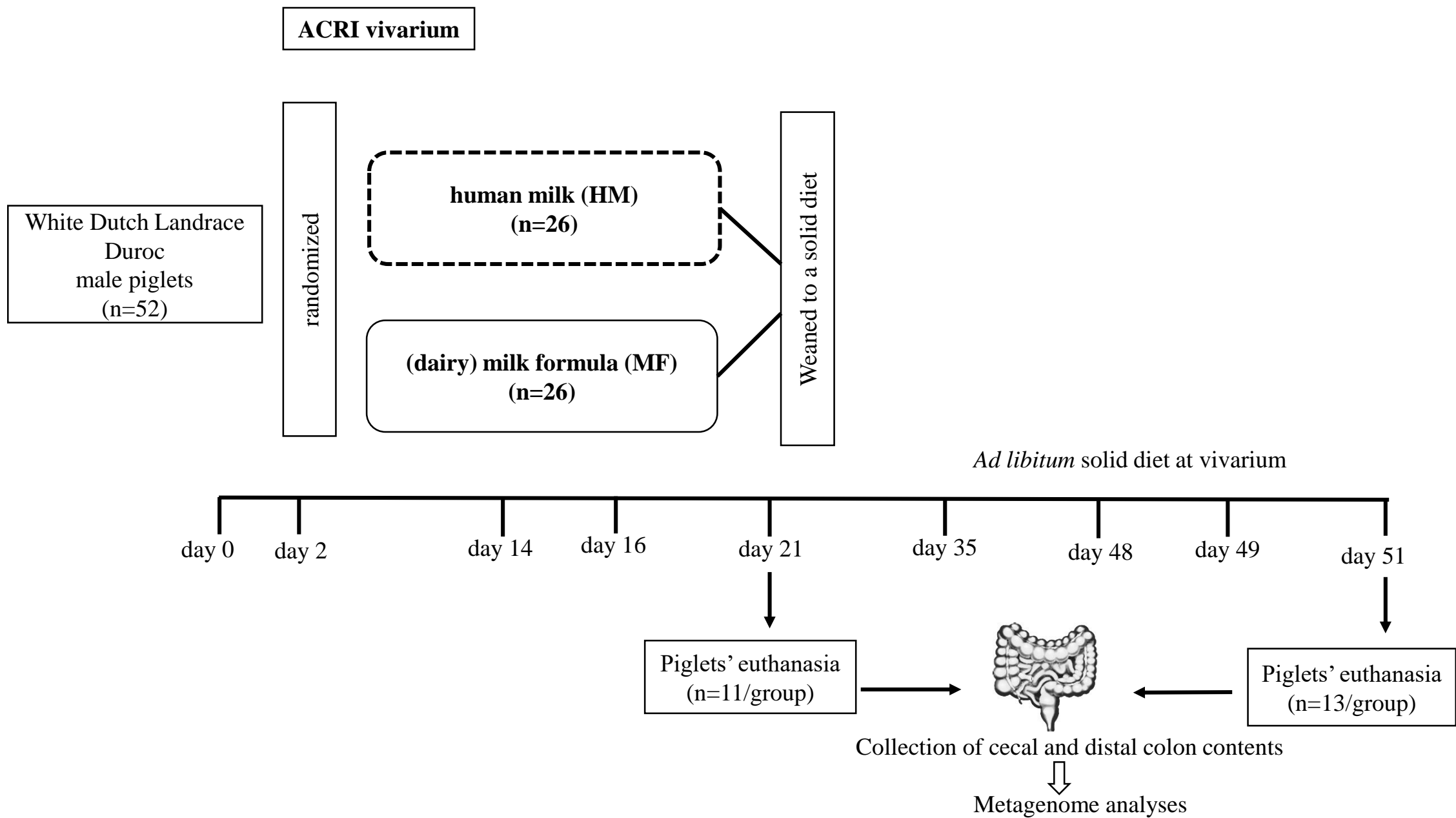

Supplement: Supplementary Figure 1 — Flowchart of the experimental design used in this study. Piglets were randomly assigned to consume human milk or an isocaloric dairy milk–based formula, trained to drink from rubber nipples and were fed 1.047 MJ kg–1 d–1 of either human milk or milk formula. Piglets were fed every 2 h in the first week of the study, followed by every 4 h in the second week, and every 6 h in the third week through day 21. Solid “starter pig food” was slowly introduced at day 14 until day 21 and all piglets had transitioned completely to an ad libitum solid diet (Teklad diet, TD 140608; Harlan) at day 21. Piglet weights and diet consumption were recorded daily. [file Data_Sheet_1.PDF]

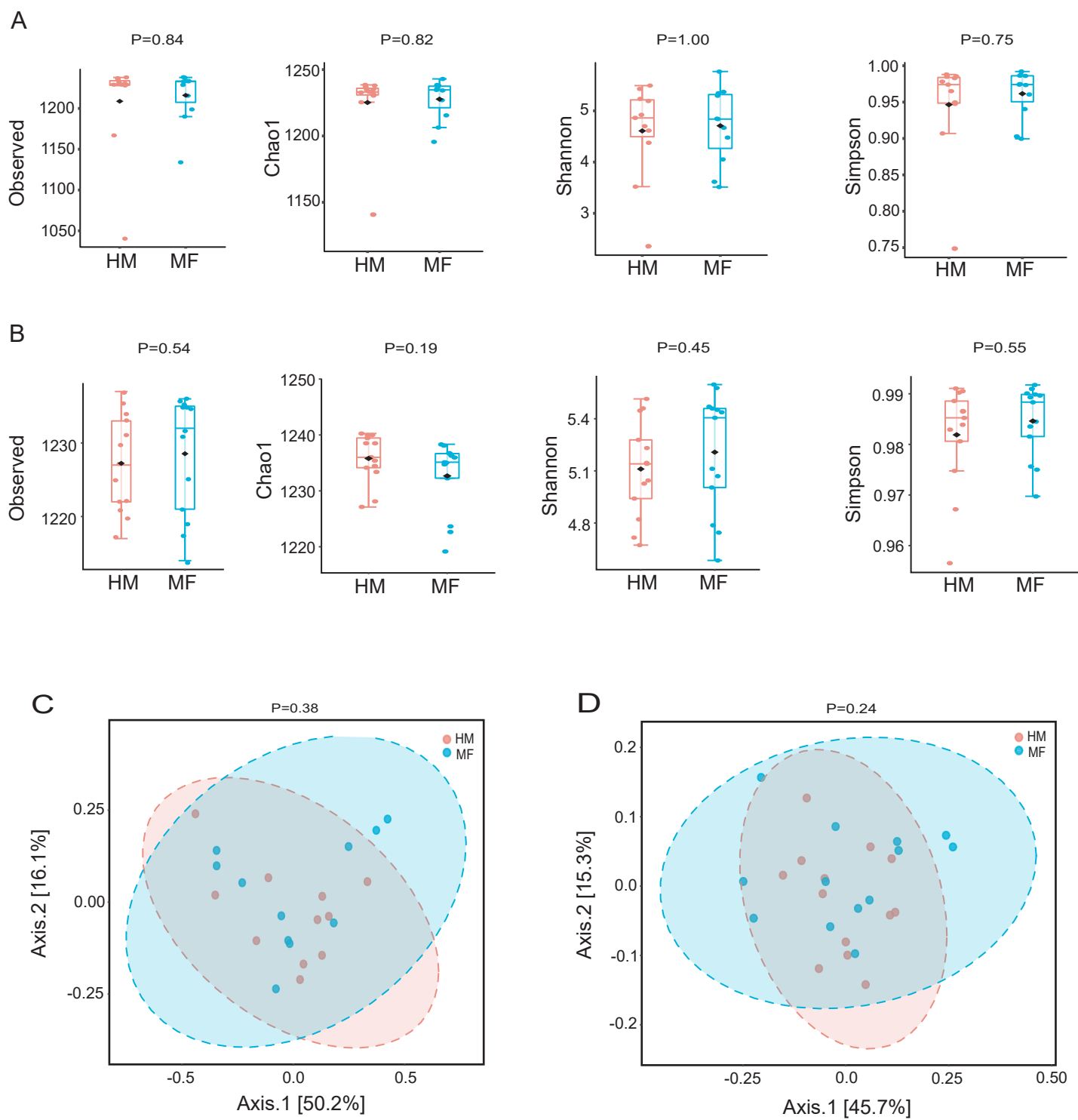

Supplement: Supplementary Figure 2 — Bacterial species at PND 21 in piglets fed with human milk (HM) or milk formula (MF). (A) Cecal alpha diversity represented by Observed, Chao1, Shannon, and Simpson indices. (B) Distal colon alpha diversity represented by Observed, Chao1, Shannon, and Simpson indices. (C) Cecal beta diversity determined by principal coordinate analysis (PCoA). (D) Distal colon beta diversity determined by PCoA. [file Data_Sheet_2.PDF]

A

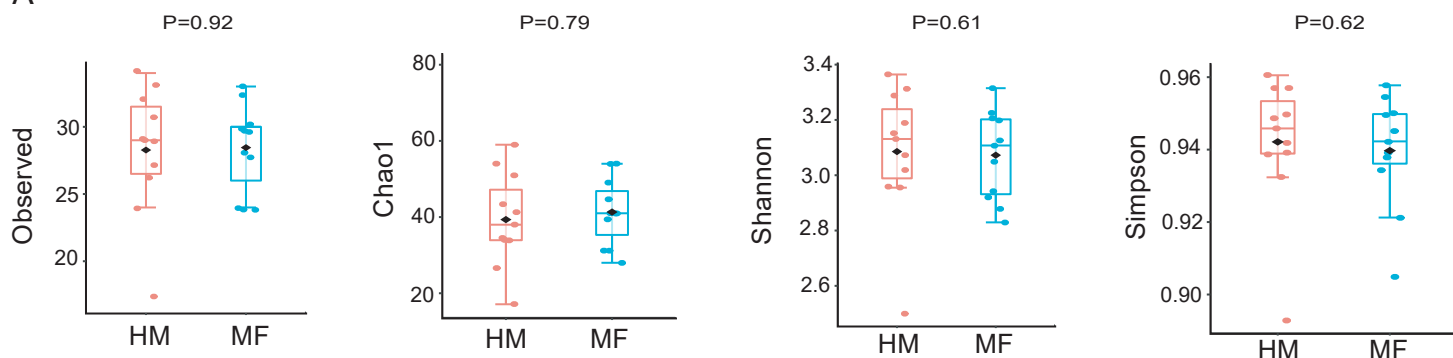

B

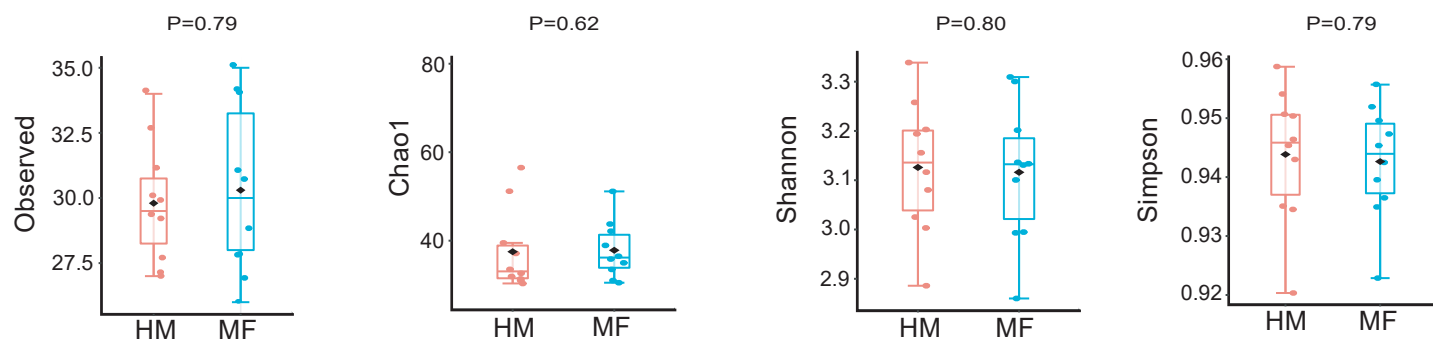

C

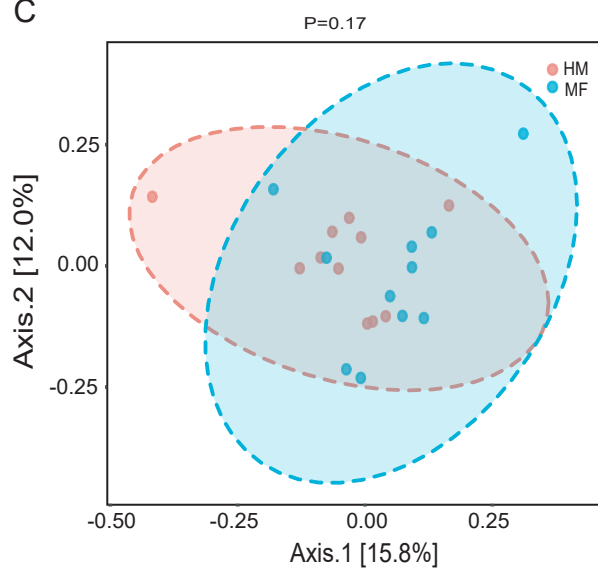

D

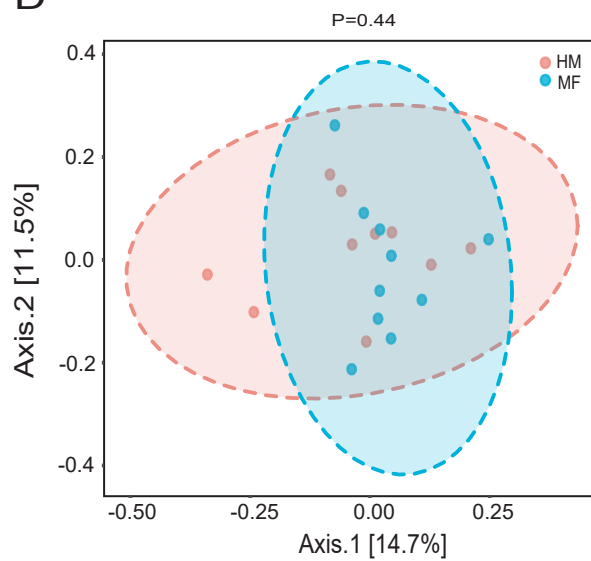

Supplement: Supplementary Figure 3 — Fungal species at PND 21 in piglets fed with human milk (HM) or milk formula (MF). (A) Cecal alpha diversity represented by Observed, Chao1, Shannon, and Simpson indices. (B) Distal colon alpha diversity represented by Observed, Chao1, Shannon, and Simpson indices. (C) Cecal beta diversity determined by principal coordinate analysis (PCoA). (D) Distal colon beta diversity determined by PCoA. [file Data_Sheet_3.PDF]

A

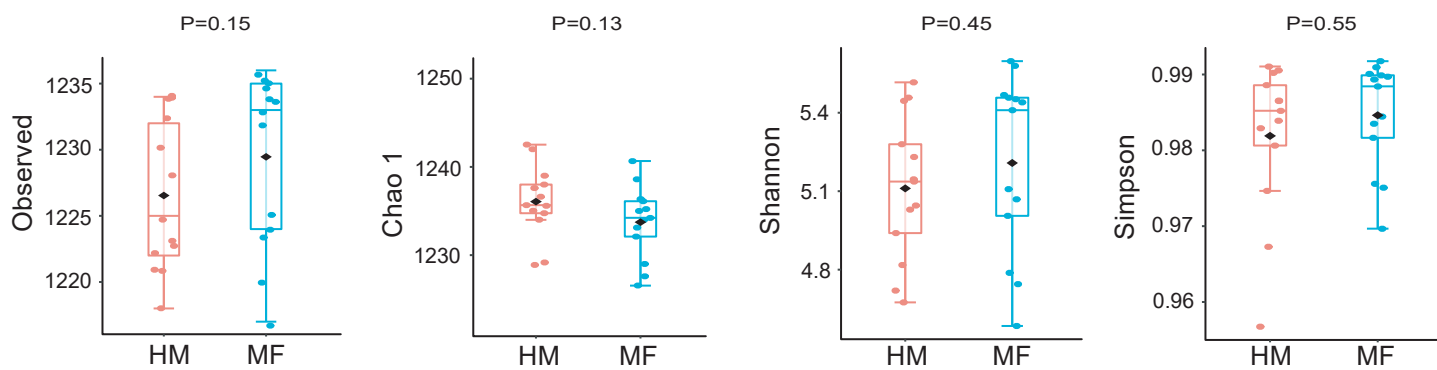

B

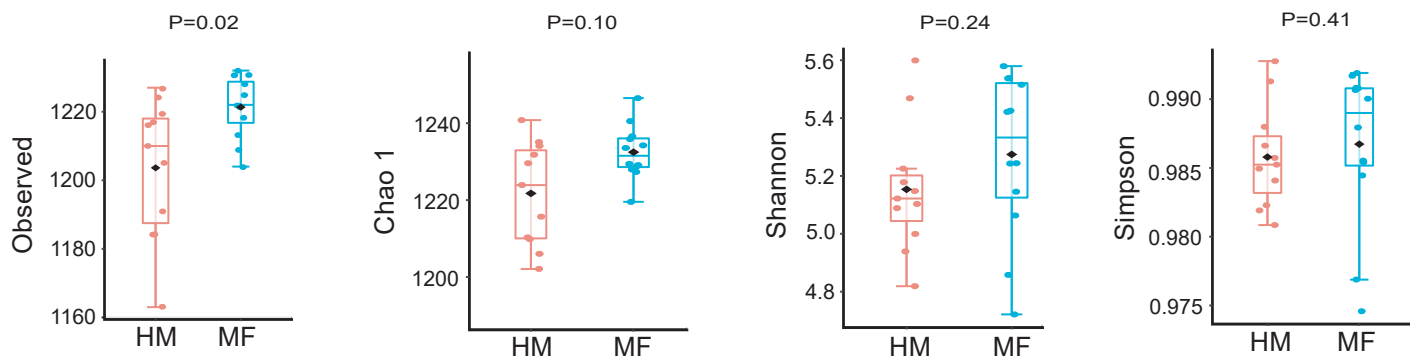

C

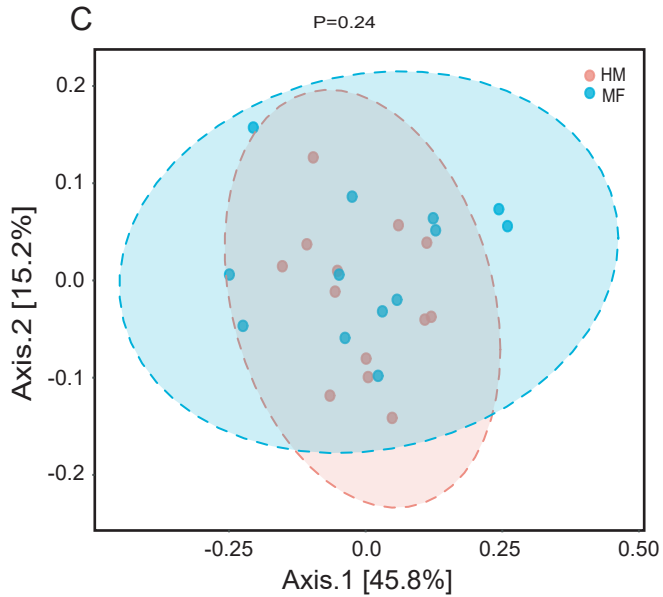

D

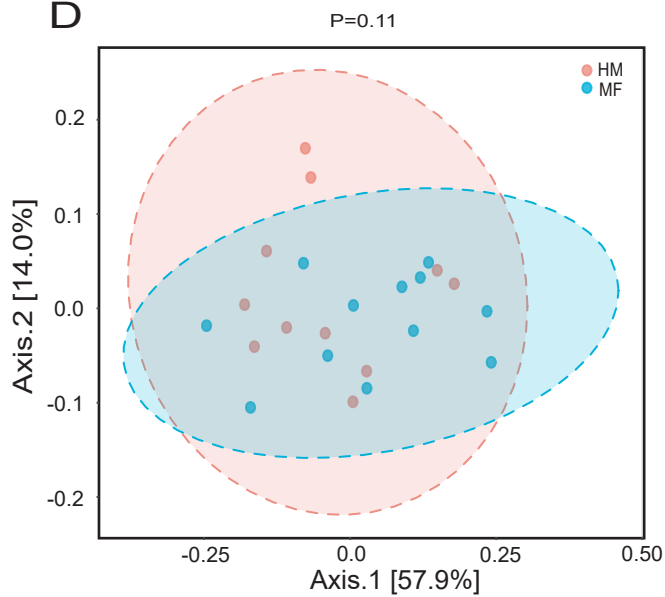

Supplement: Supplementary Figure 4 — Bacterial species at PND 51 in piglets fed with human milk (HM) or milk formula (MF). (A) Cecal alpha diversity represented by Observed, Chao1, Shannon, and Simpson indices. (B) Distal colon alpha diversity represented by Observed, Chao1, Shannon, and Simpson indices. (C) Cecal beta diversity determined by principal coordinate analysis (PCoA). (D) Distal colon beta diversity determined by PCoA. [file Data_Sheet_4.PDF]

A

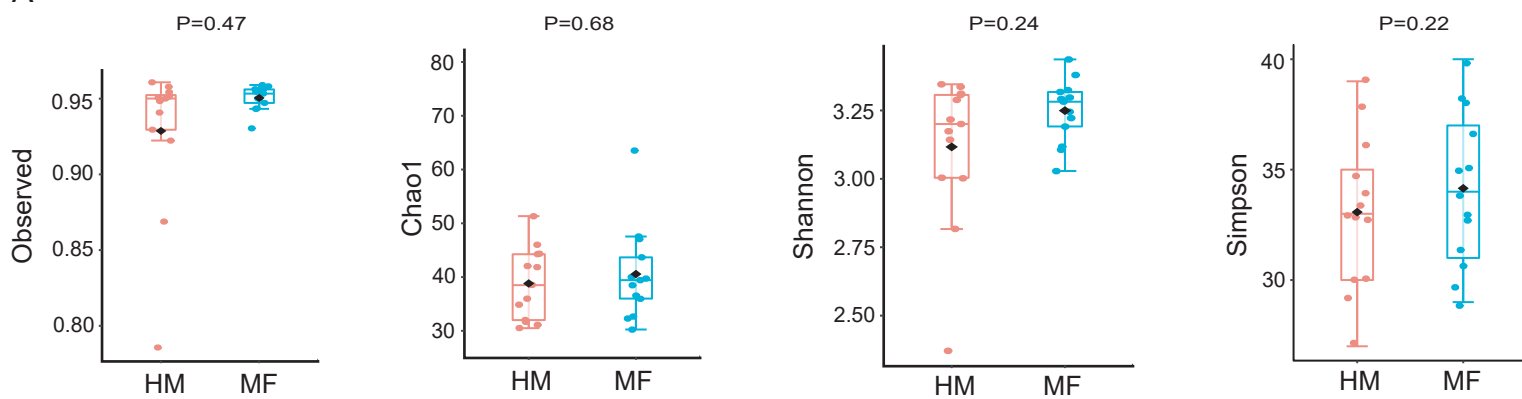

B

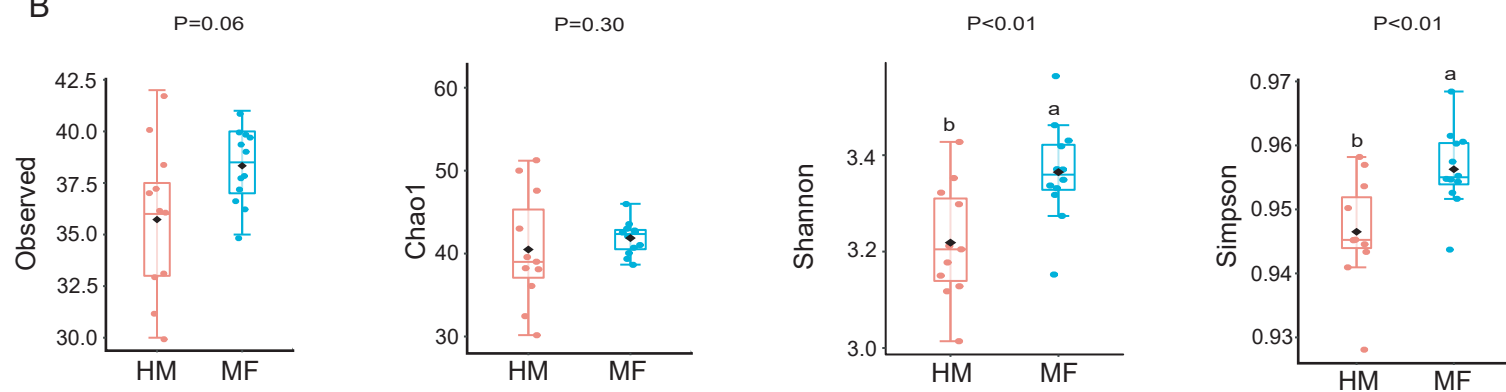

C

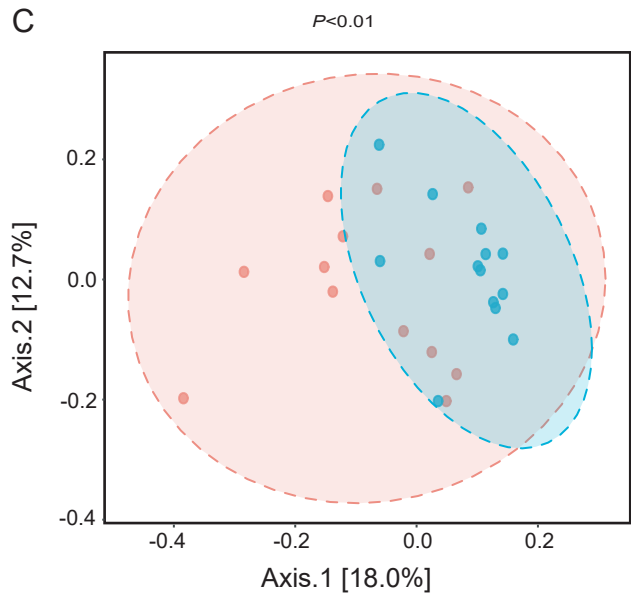

D

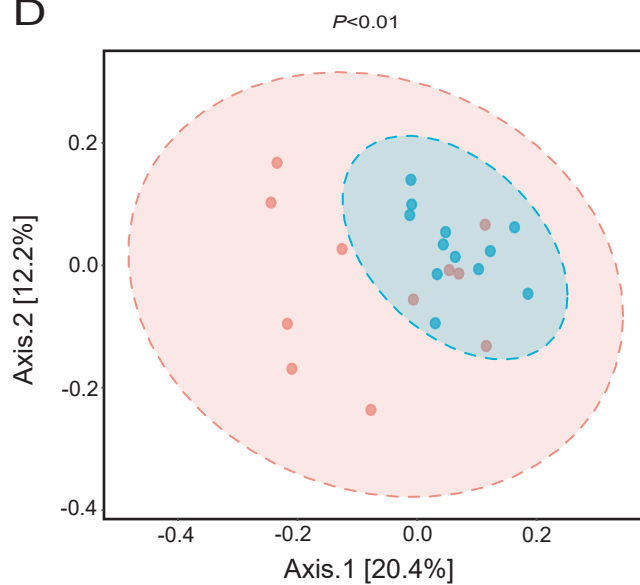

Supplement: Supplementary Figure 5 — Fungal species at PND 51 in piglets fed with human milk (HM) or milk formula (MF). (A) Cecal alpha diversity represented by Observed, Chao1, Shannon, and Simpson indices. (B) Distal colon alpha diversity represented by Observed, Chao1, Shannon, and Simpson indices. (C) Cecal beta diversity determined by principal coordinate analysis (PCoA). (D) Distal colon beta diversity determined by PCoA. [file Data_Sheet_5.PDF]
