# Supplementary material for: Bacterial and Fungal Adaptations in Cecum and Distal Colon of Piglets Fed With Dairy-Based Milk Formula in Comparison With Human Milk
Source: Front Microbiol. 2022 Mar 23;13:801854. doi: 10.3389/fmicb.2022.801854 (PMC8989072; doi:10.3389/fmicb.2022.801854)
Supplement: Supplementary Figure 6 — CAZymes percent abundance in piglets fed with human milk (HM) or milk formula (MF). (A) Cecal abundance at PND 21. (B) Cecal abundance at PND 51. (C) Colon abundance at PND 21. (D) Colon abundance at PND 51. [file Data_Sheet_6.PDF]

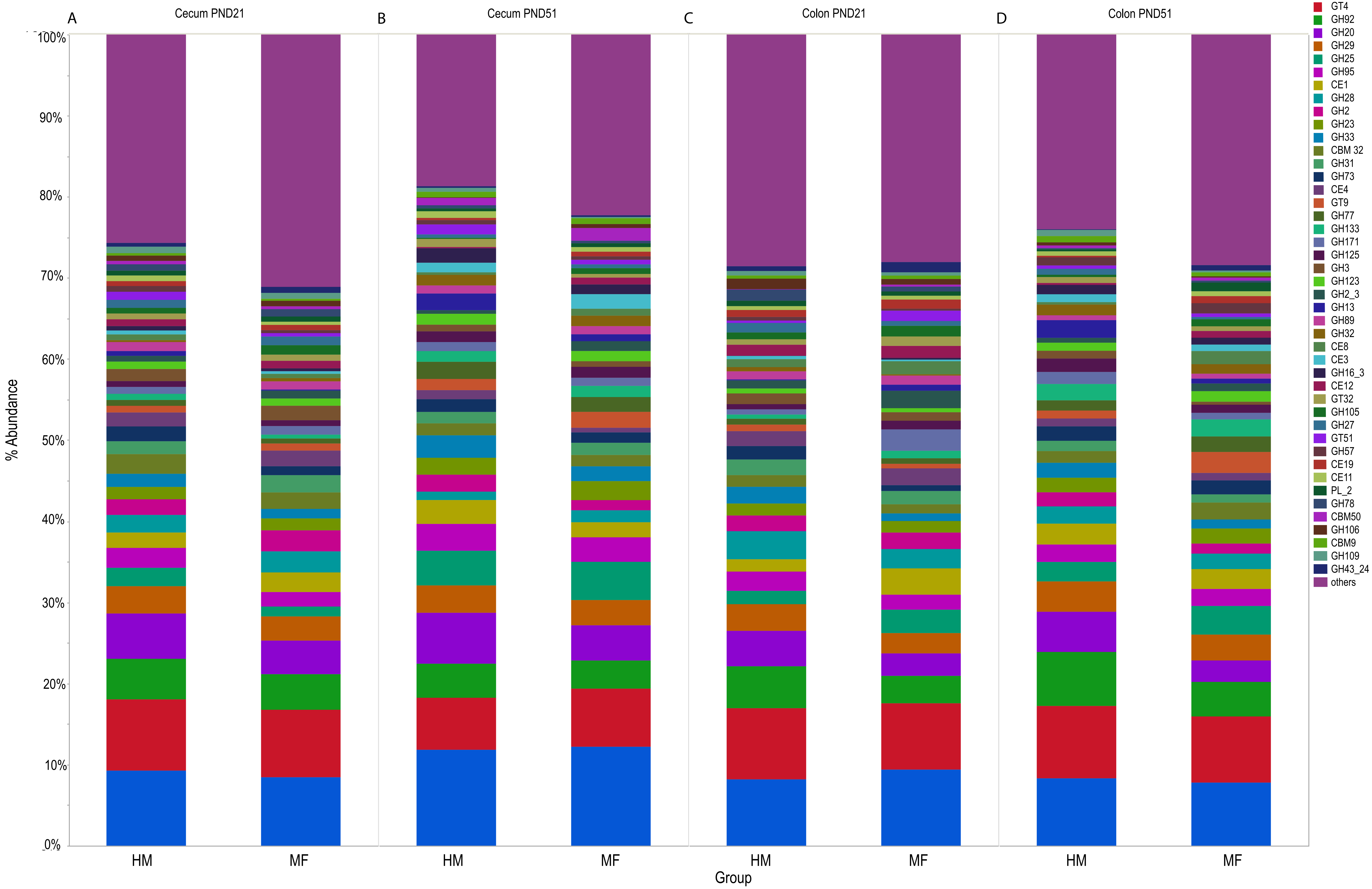
A
Cecum PND21
B
Cecum PND51
C
Colon PND21
D
Colon PND51
% Abundance
100%
90%
80%
70%
60%
50%
40%
30%
20%
10%
0%
HM
MF
HM
MF
HM
MF
HM
MF
Group
GT2
GT4
GH92
GH20
GH29
GH25
GH95
CE1
GH28
GH2
GH23
GH33
CBM 32
GH31
GH73
CE4
GT9
GH77
GH133
GH171
GH125
GH3
GH123
GH2_3
GH13
GH89
GH32
CE8
CE3
GH16_3
CE12
GT32
GH105
GH27
GT51
GH57
CE19
CE11
PL_2
GH78
CBM50
GH106
CBM9
GH109
GH43_24
others

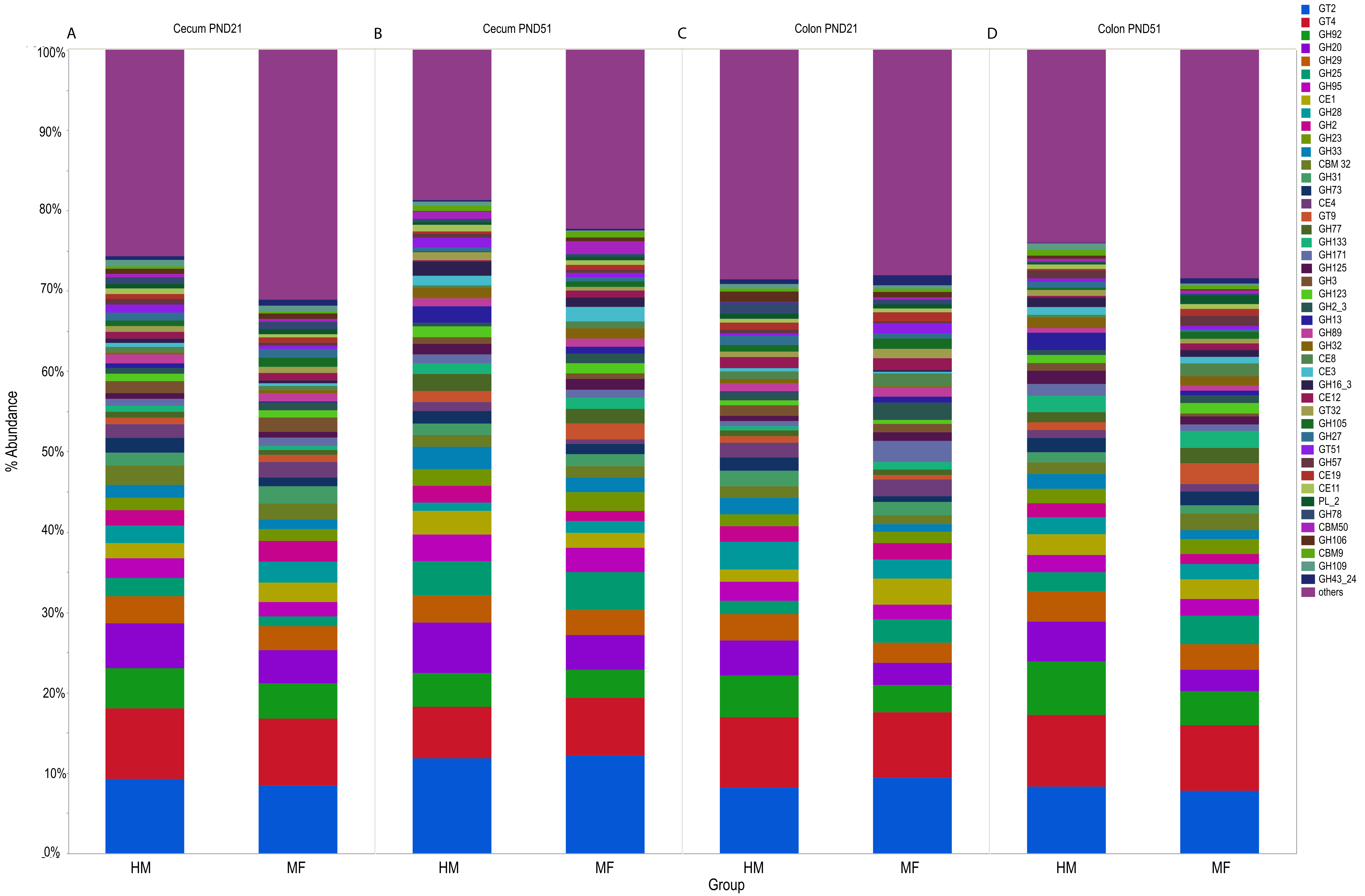

A
Cecum PND21
B
Cecum PND51
C
Colon PND21
D
Colon PND51
% Abundance
100%
90%
80%
70%
60%
50%
40%
30%
20%
10%
0%
HM
MF
HM
MF
HM
MF
HM
MF
Group
GT2
GT4
GH92
GH20
GH29
GH25
GH95
CE1
GH28
GH2
GH23
GH33
CBM 32
GH31
GH73
CE4
GT9
GH77
GH133
GH171
GH125
GH3
GH123
GH2_3
GH13
GH89
GH32
CE8
CE3
GH16_3
CE12
GT32
GH105
GH27
GT51
GH57
CE19
CE11
PL_2
GH78
CBM50
GH106
CBM9
GH109
GH43_24
others

GT2
GT4
GH92
GH20
GH29
GH25
GH95
CE1
GH28
GH2
GH23
GH33
CBM 32
GH31
GH73
CE4
GT9
GH77
GH133
GH171
GH125
GH3
GH123
GH2_3
GH13
GH89
GH32
CE8
CE3
GH16_3
CE12
GT32
GH105
GH27
GT51
GH57
CE19
CE11
PL_2
GH78
CBM50
GH106
CBM9
GH109
GH43_24
others
