# Supplementary material for: Bacterial and Fungal Adaptations in Cecum and Distal Colon of Piglets Fed With Dairy-Based Milk Formula in Comparison With Human Milk
Source: Front Microbiol. 2022 Mar 23;13:801854. doi: 10.3389/fmicb.2022.801854 (PMC8989072; doi:10.3389/fmicb.2022.801854)
Supplement: Supplementary file 7 [file Data_Sheet_7.zip › Table 2.DOCX]

**Supplementary Table 2**: Relative abundances of cecum-associated bacterial and fungal phyla detected at weaning (i.e., day 21 of age) in male piglets fed human milk (HM) or milk formula (MF) during the preweaning period from day 2 until day 21 of age.

| **Cecal Bacterial Phyla** | | | |
| --- | --- | --- | --- |
|  | **Mean % abundance ± SEM** | |  |
| **Phyla** | **HM** | **MF** | ***P* value^a^** |
| Bacteroidetes | 57.802 ± 6.611 | 54.966 ± 8.024 | 0.95 |
| Firmicutes | 26.375 ± 4.427 | 30.922 ± 6.515 | 1.00 |
| Proteobacteria | 7.046 ± 1.115 | 6.994 ± 1.059 | 1.00 |
| Verrucomicrobia | 3.821 ± 2000 | 1.476 ± 0.886 | 0.44 |
| Actinobacteria | 2.070 ± 0.403 | 2.674 ± 0.568 | 0.48 |
| Spirochaetes | 0.612 ± 0.176 | 0.494 ± 0.135 | 0.56 |
| Fusobacteria | 0.390 ± 0.064 | 0.546 ± 0.115 | 0.40 |
| Cyanobacteria | 0.313 ± 0.043 | 0.311 ± 0.061 | 0.75 |
| Synergistetes | 0.199 ± 0.044 | 0.204 ± 0.043 | 1.00 |
| Chloroflexi | 0.190 ± 0.027 | 0.208 ± 0.041 | 1.00 |
| Thermotogae | 0.179 ± 0.030 | 0.207 ± 0.046 | 0.85 |
| Chlorobi | 0.164 ± 0.019 | 0.165 ± 0.038 | 0.44 |
| Lentisphaerae | 0.134 ± 0.073 | 0.088 ± 0.037 | 0.95 |
| Acidobacteria | 0.105 ± 0.016 | 0.109 ± 0.021 | 0.95 |
| Deinococcus Thermus | 0.104 ± 0.015 | 0.117 ± 0.027 | 0.74 |
| **Cecal Fungal Phyla** | | | |
| Ascomycota | 85.403 ± 1.854 | 82.202 ± 2.344 | 0.39 |
| Basidiomycota | 14.597 ± 1.854 | 17.798 ± 2.344 | 0.29 |

^a^*P*-values were determined by Mann-Whitney test.
