# Supplementary material for: Bacterial and Fungal Adaptations in Cecum and Distal Colon of Piglets Fed With Dairy-Based Milk Formula in Comparison With Human Milk
Source: Front Microbiol. 2022 Mar 23;13:801854. doi: 10.3389/fmicb.2022.801854 (PMC8989072; doi:10.3389/fmicb.2022.801854)
Supplement: Supplementary file 7 [file Data_Sheet_7.zip › Table 3.DOCX]

**Supplementary Table 3**: Relative abundances of distal colon-associated bacterial and fungal phyla detected at weaning (i.e., day 21 of age) in male piglets fed human milk (HM) or milk formula (MF) during the preweaning period from day 2 until day 21 of age.

| **Distal Colon Bacterial Phyla** | | | |
| --- | --- | --- | --- |
|  | **Mean % abundance ± SEM** | |  |
| **Phyla** | **HM** | **MF** | ***P* value^a^** |
| *Bacteroidetes* | 56.924 ± 2.829 | 55.615 ± 4.19 | 0.65 |
| *Firmicutes* | 32.995 ± 2.725 | 31.833 ± 3.535 | 1.00 |
| *Proteobacteria* | 4.688 ± 0.433 | 6.595 ± 0.600 | 0.01 |
| *Actinobacteria* | 2.053 ± 0.157 | 2.191 ± 0.208 | 0.65 |
| *Lentisphaerae* | 0.560 ± 0.301 | 0.357 ± 0.164 | 0.92 |
| *Spirochaetes* | 0.487 ± 0.063 | 0.539 ± 0.065 | 0.45 |
| *Fusobacteria* | 0.409 ± 0.031 | 0.451 ± 0.041 | 0.39 |
| *Cyanobacteria* | 0.256 ± 0.015 | 0.428 ± 0.078 | 0.06 |
| *Chloroflexi* | 0.189 ± 0.013 | 0.223 ± 0.022 | 0.26 |
| *Synergistetes* | 0.185 ± 0.012 | 0.227 ± 0.021 | 0.09 |
| *Verrucomicrobia* | 0.178 ± 0.019 | 0.325 ± 0.148 | 0.52 |
| *Chlorobi* | 0.175 ± 0.011 | 0.201 ± 0.013 | 0.08 |
| *Thermotogae* | 0.167 ± 0.012 | 0.180 ± 0.018 | 0.58 |
| *Fibrobacteres* | 0.161 ± 0.009 | 0.173 ± 0.011 | 0.36 |
| **Distal Colon Fungal Phyla** | | | |
| *Ascomycota* | 83.483 ± 1.873 | 85.955 ± 1.222 | 0.34 |
| *Basidiomycota* | 16.517 ± 1.873 | 14.045 ± 1.222 | 0.34 |

^a^*P*-values were determined by Mann-Whitney test.
