# Supplementary material for: Bacterial and Fungal Adaptations in Cecum and Distal Colon of Piglets Fed With Dairy-Based Milk Formula in Comparison With Human Milk
Source: Front Microbiol. 2022 Mar 23;13:801854. doi: 10.3389/fmicb.2022.801854 (PMC8989072; doi:10.3389/fmicb.2022.801854)
Supplement: Supplementary file 7 [file Data_Sheet_7.zip › Table 4.DOCX]

**Supplementary Table 4**: Relative abundances of cecum-associated bacterial and fungal phyla detected postweaning (i.e., day 51 of age) in male piglets fed human milk (HM) or milk formula (MF) during the preweaning period from day 2 until day 21 of age.

| **Cecal Bacterial Phyla** | | | |
| --- | --- | --- | --- |
|  | **Mean % abundance ± SEM** | |  |
| **Phyla** | **HM** | **MF** | ***P* value^a^** |
| *Bacteroidetes* | 56.917 ± 2.828 | 55.604 ± 4.193 | 0.65 |
| *Firmicutes* | 33.006 ± 2.729 | 31.823 ± 3.526 | 1.00 |
| *Proteobacteria* | 4.685 ± 0.432 | 6.595 ± 0.602 | 0.01 |
| *Actinobacteria* | 2.056 ± 0.155 | 2.217 ± 0.213 | 0.48 |
| *Lentisphaerae* | 0.562 ± 0.301 | 0.358 ± 0.165 | 1.00 |
| *Spirochaetes* | 0.483 ± 0.063 | 0.533 ± 0.064 | 0.48 |
| *Fusobacteria* | 0.415 ± 0.032 | 0.456 ± 0.041 | 0.42 |
| *Cyanobacteria* | 0.256 ± 0.016 | 0.422 ± 0.079 | 0.09 |
| *Synergistetes* | 0.188 ± 0.012 | 0.228 ± 0.022 | 0.12 |
| *Chloroflexi* | 0.187 ± 0.012 | 0.224 ± 0.022 | 0.21 |
| *Verrucomicrobia* | 0.180 ± 0.019 | 0.322 ± 0.148 | 0.65 |
| *Chlorobi* | 0.174 ± 0.011 | 0.203 ± 0.013 | 0.08 |
| *Thermotogae* | 0.164 ± 0.012 | 0.180 ± 0.017 | 0.42 |
| *Fibrobacteres* | 0.160 ± 0.008 | 0.171 ± 0.011 | 0.42 |
| **Cecal Fungal Phyla** | | | |
| *Ascomycota* | 86.309 ± 1.607 | 83.204 ± 1.291 | 0.16 |
| *Basidiomycota* | 13.691 ± 1.607 | 16.796 ± 1.291 | 0.16 |

^a^*P*-values were determined by Mann-Whitney test.
