# Supplementary material for: Bacterial and Fungal Adaptations in Cecum and Distal Colon of Piglets Fed With Dairy-Based Milk Formula in Comparison With Human Milk
Source: Front Microbiol. 2022 Mar 23;13:801854. doi: 10.3389/fmicb.2022.801854 (PMC8989072; doi:10.3389/fmicb.2022.801854)
Supplement: Supplementary file 7 [file Data_Sheet_7.zip › Table 5.DOCX]

**Supplementary Table 5**: Relative abundances of colon-associated bacterial and fungal phyla detected at postweaning (i.e., day 51 of age) in male piglets fed human milk (HM) or milk formula (MF) during the preweaning period from day 2 until day 21 of age.

| **Distal Colon Bacterial Phyla** | | | |
| --- | --- | --- | --- |
|  | **Mean % abundance ± SEM** | |  |
| **Phyla** | **HM** | **MF** | ***P* value^a^** |
| *Bacteroidetes* | 58.428 ± 3.22 | 51.708 ± 4.14 | 0.21 |
| *Firmicutes* | 33.397 ± 2.533 | 37.428 ± 3.527 | 0.35 |
| *Proteobacteria* | 3.697 ± 0.272 | 5.235 ± 0.442 | <0.01 |
| *Actinobacteria* | 1.547 ± 0.132 | 2.068 ± 0.144 | 0.02 |
| *Spirochaetes* | 0.485 ± 0.085 | 0.757 ± 0.113 | 0.04 |
| *Fusobacteria* | 0.391 ± 0.036 | 0.468 ± 0.033 | 0.10 |
| *Lentisphaerae* | 0.277 ± 0.126 | 0.139 ± 0.025 | 0.88 |
| *Cyanobacteria* | 0.230 ± 0.017 | 0.310 ± 0.024 | 0.01 |
| *Chloroflexi* | 0.200 ± 0.025 | 0.251 ± 0.020 | 0.07 |
| *Chlorobi* | 0.177 ± 0.009 | 0.197 ± 0.011 | 0.28 |
| *Verrucomicrobia* | 0.167 ± 0.010 | 0.192 ± 0.021 | 0.69 |
| *Thermotogae* | 0.162 ± 0.016 | 0.196 ± 0.016 | 0.18 |
| *Fibrobacteres* | 0.160 ± 0.007 | 0.188 ± 0.010 | 0.04 |
| *Synergistetes* | 0.157 ± 0.018 | 0.226 ± 0.020 | 0.02 |
| **Distal Colon Fungal Phyla** | | | |
| *Ascomycota* | 89.911 ± 0.811 | 85.264 ± 1.13 | 0.01 |
| *Basidiomycota* | 10.089 ± 0.811 | 14.736 ± 1.13 | 0.01 |

^a^*P*-values were determined by Mann-Whitney test.
